# Robust and versatile assembly for emitter positioning, observation, and heating in atmospheric pressure field desorption mass spectrometry

**DOI:** 10.1177/14690667241236073

**Published:** 2024-03-15

**Authors:** Jan Schweinfurth, H. Bernhard Linden, Jürgen H. Gross

**Affiliations:** 1 Institute of Inorganic Chemistry, Heidelberg University, Heidelberg, Germany; 2 Linden CMS, Weyhe, Germany; 3 Institute of Organic Chemistry, Heidelberg University, Heidelberg, Germany

**Keywords:** atmospheric pressure field desorption, field desorption, field ionization, emitter heating current, activated field emitter, Fourier transform-ion cyclotron resonance, ion source, ambient desorption/ionization (ADI), trapped ion mobility-quadrupole-time-of-flight (TIMS-Q-TOF)

## Abstract

Atmospheric pressure field desorption (APFD) mass spectrometry (MS) has recently been introduced as a new variant of field desorption (FD) mass spectrometry. The development aimed at providing the basic characteristics of FD-MS in combination with instruments equipped with an atmospheric pressure (AP) interface. Hitherto, APFD has been demonstrated to yield both positive and negative even electron ions of highly polar or ionic compounds as well as to enable the generation of positive molecular ions, M^+•^, of polycyclic aromatic compounds. The prototype setup for APFD was based on a nano-electrospray ionization (nanoESI) source slightly modified to allow for emitter positioning in front of the AP interface of a Fourier transform-ion cyclotron resonance (FT-ICR) mass spectrometer. The entrance electrode of the interface was set to negative or positive high voltage with respect to the emitter at ground potential, thereby permitting the formation of positive or negative ions, respectively. This work describes a custom-built device for quicker and more reproducible sample loading on and positioning of field emitters at the entrance electrode of the atmospheric pressure interface of a mass spectrometer. In addition, the device provides means for observation of the emitter during operation and for resistive emitter heating as employed in traditional FD-MS. Emitter heating both speeds up the desorption of the analytes and allows for the desorption/ionization of analytes of higher molecular weight than without emitter heating. In some cases, the signal-to-noise ratio of APFD mass spectra is improved due to higher ion currents effected by compressing the entire process into shorter periods of spectral acquisition. The new setup enables robust and reliable operation in APFD-MS. Moreover, it has been designed as to allow for use on a range of instruments as it can either be used on an FT-ICR mass spectrometer or in combination with a trapped ion mobility-quadrupole-time-of-flight (TIMS-Q-TOF) instrument.

## Introduction

Field ionization (FI) and soon after field desorption (FD) have been developed in the late 1960s and early 1970s to enable soft ionization in mass spectrometry (MS).^1–4^ In case of compounds of low polarity, FI and FD normally yield intact positive molecular ions, M^+•^, while highly polar compounds form protonated molecules, [M + H]^+^, and/or alkali ion adducts, [M + alkali]^+^.^1–4^ Ionization via the FI pathway relies on electric fields in the order of 1–2 V Å^–1^ that can effect tunneling of an electron from the neutral analyte molecule towards a positive electrode represented by the so-called field emitter.^1,5,6^ FD of preformed ions may occur at field strengths that are about two orders of magnitude lower.^5,7–11^ To effectively deliver M^+•^ ion by the FI process, the very high electric field strengths are achieved by using activated field emitters.^12–14^ Those activated tungsten wire emitters provide thousands of field-enhancing microneedles that also serve as a large surface for deposition of the sample.

Being a desorption/ionization method, FD particularly expanded the range of compound classes accessible to MS. Further innovation came along with liquid-injection field desorption/ionization (LIFDI),^15,16^ because LIFDI permitted sample application to the emitter under the complete exclusion of moisture and air.^15–19^ Notwithstanding the numerous advancements in FI, FD, and LIFDI, these were exclusively performed in high vacuum, in particular as FI and FD had matured in the realm of magnetic sector instruments.^1–3^ Later, FI, FD, and LIFDI were transferred to modern mass analyzers like Fourier transform ion cyclotron resonance (FT-ICR)^20–24^ and orthogonal-acceleration time-of-flight (oaTOF).^25–30^

On one hand, during the last two decades mass spectrometers equipped with atmospheric pressure (AP) interfaces became dominant while fewer modern instruments were available that were suited for vacuum ionization techniques. On the other hand, the introduction of ambient desorption/ionization (ADI) techniques initiated the development of a plethora of ambient MS methods, some of which became highly successful.^31–33^ Around the development of the most prominent ambient MS techniques, the exploration of a large range of techniques was initiated, some of which using fibers, wires, needles, and other sharp objects in combination with strong electric fields to effect desorption/ionization. Among these, those with some relationship to FD are the use of a sharp stainless steel needle in front of an API interface,^34^ field-induced wooden tip electrospray ionization,^35^ carbon fiber ionization,^36^ the general use of an insulating fiber as sampling probe and ionization substrate,^37^ and some others.^38–42^

Attempts have been made to permit FD in a non-vacuum environment, preferably at atmospheric pressure. This led to the development of superatmospheric pressure FD where emitter potentials in the order of 10 kV can be used while electric discharges to the counter electrode are still suppressed.^5^ This way, positive ions of various ionic and highly polar compounds were generated from bare tungsten wire emitters set to 9–12 kV relative to a counter electrode and FD spectra exhibiting intensive signals were obtained.^5^ Trials with activated FD emitters at atmospheric pressure were scarce and their application rather was meant as a control experiment like in a study testing various natural microscale emitters like hairy legs of *Drosophila* flies.^43^ There, activated emitters were used as a reference, and in fact, [M + H]^+^ ions of hexakis-(fluoroalkoxy)-phosphazenes from a commercial mass calibration mixture^44,45^ were detected.^43^ Activated tungsten field emitters at atmospheric pressure were also used in a study exploring the effect of very strong electric field on reaction rates. This revealed in three cases, that when standard 13-µm activated tungsten wire emitters were used for FD at the open atmosphere, increased reaction rates were observed.^6^

All of those publications inspired the recent exploration of atmospheric pressure field desorption (APFD) using standard activated 13-µm tungsten wire emitters.^46^ There, the formation of positive and also of negative even electron ions has been reported for ionic or at least highly polar analytes. In general, probing the feasibility of FD under ambient conditions appeared to be worthwhile.^46^ Soon after, it was demonstrated that APFD could even form molecular ions, M^+•^, of various polycyclic aromatic compounds via field ionization.^47^ Most recently, the application of negative-ion APFD for the analysis of surfactants in commercial detergents was presented to demonstrate the use of APFD with some real-world samples.^48^

During that period, it often appeared that a more robust and reproducible means of positioning and aligning the emitter would notably improve the practical use of APFD, in particular, as conducting experiments that needed a number of repetitions was sometimes demanding.^48^

The original and rather basic setup based on a commercial nanoESI source was hampered by the fact that it did not align the emitter in parallel to the rim of the opening of the spray shield electrode. Instead the emitter approached the upper rim by about 0.5 mm closer than the lower end. Moreover, due to the simple screw-in nature of the emitter clamp, the emitter was not always in the same vertical orientation and swapping emitters could also result in slight shifts along the slot axis of the clamp.^46^

Ideally, an emitter should be positioned at exactly the same position, regardless of whether it has just been exchanged or retracted for loading or rinsing or whether even the entire source assembly has freshly been mounted to the instrument. The aim of the work presented here was to construct and test such an emitter holding assembly for APFD. Additionally, it should implement resistive emitter heating as in traditional FD^49–51^ plus a microscope USB camera to observe the emitter during loading and operation.

## Experimental

### Mass spectrometer

A Bruker Apex-Qe Fourier transform ion cyclotron resonance (FT-ICR) mass spectrometer (Bruker Daltonics, Bremen, Germany) was employed for most of the APFD experiments. The instrument was equipped with a 9.4 T superconducting magnet and an ESI-to-MALDI switchable Dual Source. The mass spectrometer was controlled by the Bruker ApexControl software (V 3.0.0) and data analysis was performed using the Bruker DataAnalysis software (V 4.3).

External mass calibrations were established in ESI mode by either using Agilent Tune Mix (G1969-85000) for the *m/z* 200–1800 range^44,45^ or arginine [Arg_n_–H]^–^ cluster ions for the *m/z* 150–1200 range.^52,53^ Mass accuracy was generally in the order of 3 ppm.

### Description of the APFD assembly

**Figure 1.**
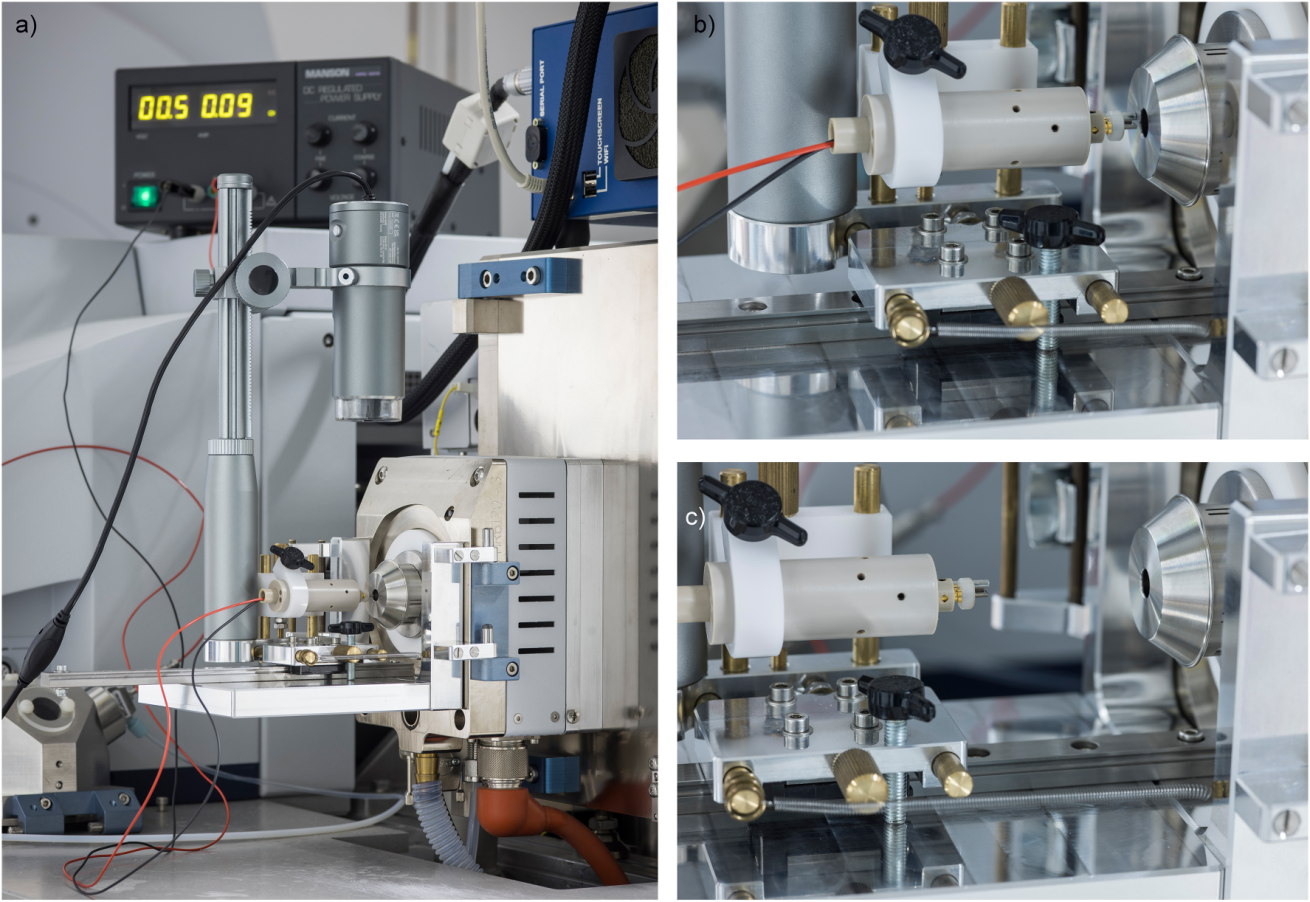
Photographs of **a)** the entire APFD source assembly mounted to the AP interface of the Bruker ApexQe FT-ICR mass spectrometer with the DC power supply for the EHC placed on top of the instrument in the background (showing 0.09 A at 0.5 V), **b)** the probe tip with emitter positioned at the spray shield electrode as in operation, and **c)** the probe tip in retracted position for emitter loading or swapping.

The APFD assembly is based on an aluminium frame machined to fit the AP interface of the Bruker ApexQe FT-ICR mass spectrometer, i.e., the hinges are screwed on to the right side of the frame while the holding for the source door clip is screwed on to the left (Figure 1). The frame supports a rail at the bottom along which an *x*,*y*-adjustable mounting stage may slide along the *z*-axis, i.e., forth toward the spray shield electrode (APFD runs) and back (emitter loading or swapping). This mounting stage carries a probe holder. The APFD probe bears the emitter sockets plus holes to mount a slide-on counter electrode and provides electric feed-through for an emitter heating current (EHC) to be applied. The probe tip essentially is of the same type as used for vacuum LIFDI in combination with magnetic sector instruments (Linden CMS, Leeste, Germany). Sliding towards the counter electrode is assisted by a tension spring and the mounting stage position can be locked by tightening a star-knob screw toward the bottom of the assembly. The mounting stage also accommodates a USB microscope (DST-1028, Bresser, Rhede, Germany) enabling emitter observation from top during any phase of operation as its position is fixed with respect to the emitter. This camera plus the manufacturer's CamLabLite software were employed to document emitter loading, positioning, and heating in stills and video. As the emitter is always at ground potential, an EHC can be easily applied via a regulated power supply (MPD-6015 DC, Manson, Hong Kong, China). While Figure 1 comprises a photograph of the entire APFD assembly and of some details, design drawings are provided as Figs. S1–S4 in the Supplementary Material.

### APFD ion source operation

The first implementation of APFD and its general operation were described in detail elsewhere.^46–48^ The emitter was positioned horizontally in the center of the 8-mm wide aperture of the so-called spray shield electrode of the standard ESI source. The spray shield served as the counter electrode for APFD. Underneath, a rounded metal cap (orifice 0.90 mm in diameter) is mounted on the glass transfer capillary (orifice 0.50 mm in diameter). High voltage was exclusively applied to the counter electrode provided by the API interface while the emitter stayed at ground potential. The high voltage was set using the API source controls. At 2.0 mm distance of the emitter to the counter electrode, in this setup represented by the spray shield and capillary cap, voltages close to the upper technical limit of the source (±6 kV) normally caused spark discharges. Thus, the voltages in the range of ±3.0 to ±5.0 kV at the spray shield and ±3.5 to ±5.5 kV at the cap underneath the spray shield were employed. Both voltages were negative to attract positive ions (APFD^+^) and positive to attract negative ions (APFD^–^) and the cap voltage was generally set 0.5–1.0 kV above the spray shield voltage. Safety notice: This work describes a prototype source assembly where high voltage is applied to the uncovered spray shield. Nonetheless, basic care provided, the user is safe from getting into contact with high voltage during operation as all other parts are at ground potential.

The nebulizer gas for ESI was switched off at all times as the ESI sprayer was simply put aside without disconnecting it from the instrument during APFD. The drying gas at the spray shield was set to 1.2–2.0 l min^–1^ at 60–140 °C. Other instrument settings were the same as in ESI operation.

The samples were manually delivered to the emitter as solutions at concentrations of 1–2 mg ml^–1^ by using a 10-µl syringe while the emitter was mounted to the emitter sockets. The solvents were allowed to evaporate and the emitter was moved to the position for operation (process documented in a video in the Supplementary Material). After the runs, the emitter was either rinsed with solvent to remove excessive analyte or baked using the EHC at about 160 mA. Emitters could be used for tens of acquisitions.

The activated field emitters were of the standard type commercially available for the JEOL AccuTOF series of instruments^29,30^ and were based on 13-µm tungsten wires (Linden CMS, Weyhe, Germany).

### Acquisition of mass spectra

The ions were collected for 0.5–2.0 s per transient in FT-ICR mass analysis using the RF-only accumulation hexapole (h2). The ions were excited and detected using established APFD procedures.^46–48^ When the range *m/z* 200–1800 was selected, a 512 k data points transient resulted in a resolving power of *R* = 72,000 at about *m/z* 400, the range *m/z* 150–1200 with an 1 M data points transient delivered *R* = 115,000, respectively.

While emitter heating was not in operation a set of 16 transients was accumulated to yield a final FT-ICR mass spectrum. When an EHC ramp was used, the acquisition was set to chromatography mode where each transient (ions accumulated for 0.5–2.0 s per transient) was filed separately to allow for the selective collection of those transients where desorption occurred via data processing after the run.

### Adaptation to the timsTOFflex instrument

Finally, a Bruker timsTOFflex, i.e., a trapped ion mobility-quadrupole-time-of-flight (TIMS-Q-TOF) instrument (Bruker Daltonics, Bremen, Germany) was used to explore the adaptation of the APFD assembly to other instruments. According to the manufacturer, the AP interface of all current Bruker instruments is identical to the above, and thus, the source would be fully compatible among these. The instrument was equipped with an ESI-to-MALDI switchable Dual Source similar to that of the ApexQe instrument. The only modification of the APFD source assembly required was to swap hinges to the left and holders to the right side of the frame (Supplemental Figures S5–S8).

To circumvent swapping between ESI and APFD sources, mass calibration was done by laser desorption/ionization (LDI) of red phosphorous that yields a series of cluster ions.^54,55^ APFD operation was performed analogous to the above description, and again, other operational parameters were as in ESI mode of this instrument. The mass spectrometer was controlled by the Bruker timsControl software (V 2.0) and data analysis was performed using the Bruker DataAnalysis software (V 6.0).

### Samples

A set of samples previously explored to establish the basics of APFD mode was reused here as a control of general operation and to check for reproducibility, and thus, details on most samples were already communicated.^46–48^

The polystyrenes of average molecular weight 560 u and 1 ku were obtained from PSS Polymer Standards Service GmbH (Mainz, Germany). The samples of various types are compiled in Table 1. Solvents of LC-MS grade were obtained from Merck KGaA (Darmstadt, Germany).

**Table 1.** Compounds analyzed by APFD-MS in the order of appearance.

Compound Name	Relevant Ionic Formulas	Calculated *m/z* Value(s)
Trihexyl(tetradecyl)­phosphonium tris(pentafluoroethyl)­trifluorophosphate	[C_32_H_68_P]^+^, [C_6_F_18_P]^–^	483.5053, 444.9456
1-Aza-[6]helicene	[C_25_H_15_N]^+•^, [C_25_H_16_N]^+^	329.1199, 330.1277
Jeffamine M-2005	[CH_3_O-(C_2_H_4_O)_n_(C_3_H_6_O)_m_-NH_3_]^+^	408.3320, 466.3738, 524.4157, 582.4576, 640.4994, 698.5413, …
Benzo[a]pyrene	[C_20_H_12_]^+•^	252.0934
1,1,4,4-Tetraphenyl­butadiene	[C_28_H_22_]^+•^	358.1716
Fluoranthene	[C_16_H_10_]^+•^	202.0777
Dusy Women shower gel (containing organic sulfates and sulfonates)	Sulfates: [C_12_H_25_O_4_S]^–^, [C_14_H_29_O_5_S]^–^, [C_16_H_33_O_6_S]^–^, [C_18_H_37_O_7_S]^–^, … Sulfonates: [C_14_H_29_O_4_S]^–^, [C_16_H_33_O_5_S]^–^, [C_18_H_37_O_6_S]^–^, [C_20_H_41_O_7_S]^–^, …	265.1479, 309.1741, 353.2003, 397.2265, … 293.1792, 337.2054, 381.2316, 425.2578, …
Perfluorononanoic acid	[C_8_F_17_]^–^, [C_8_F_17_COO]^–^,	418.9734, 462.9632
Polystyrene 560	[C_4_H_10_(C_8_H_8_)_n_]^+•^	682.4533, 786.5159, 890.5785, 994.6411, …
Polystyrene 1k	[C_4_H_10_(C_8_H_8_)_n_]^+•^	682.4533, 786.5159, 890.5785, 994.6411, 1098.7037, 1202.7663, 1306.8289, 1410.8915, …

## Results and discussion

### Robustness of operation

**Figure 2.**
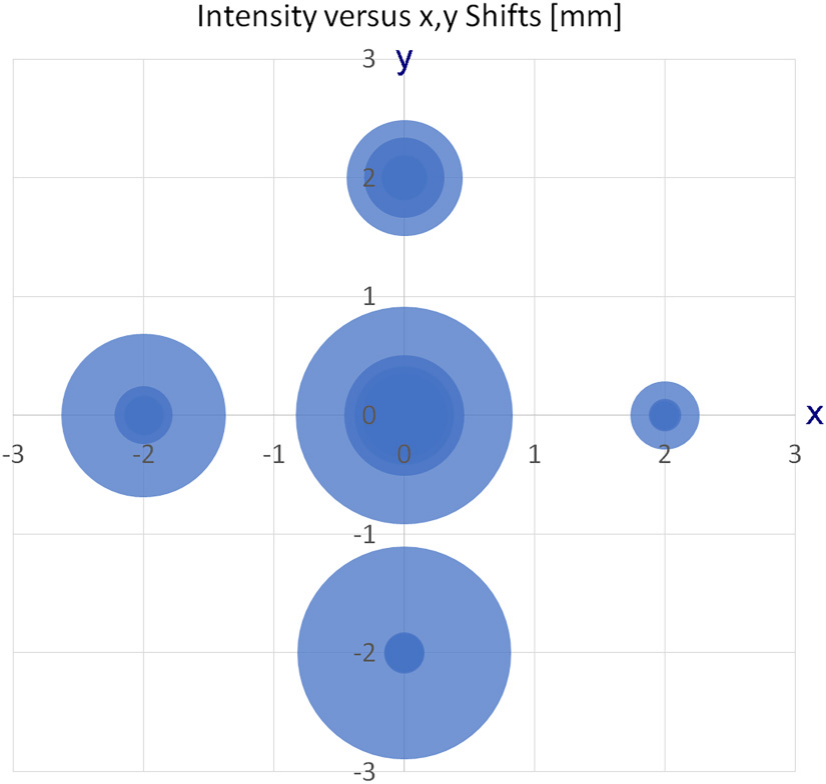
Variation of signal intensity of the ionic liquid cation [C_32_H_68_P]^+^, *m/z* 485.5037, as a function of multiple parallel shifts of the emitter in *x* and *y* directions by 2 mm in each case. Each intensity of a spectrum is represented as a circle of corresponding size.

Key to the robustness of operation of an ion source is the ease of use after having mounted the respective source. In APFD, this mainly translates into reproducibility of the emitter position and into not being susceptible to slightly off positioning of an emitter. Therefore, the emitter was intentionally misaligned by about 2 mm off the center position in either direction and spectra of the cation of the ionic liquid, [C_32_H_68_P]^+^, *m/z* 485.5037, were recorded. While the signal intensity notably dropped upon shifting that far out, the signal still persisted. The procedure was repeated and the results of several runs are summarized in (Figure 2, data in Supplemental material Figure S9). A drop of 50% in intensity, however, is only logical as a 2-mm shift already caused the emitter to partially move outside the aperture of the spray shield, particularly when shifted horizontally. This behavior demonstrates that, on one hand, good emitter alignment could quickly be achieved by visual judgment while, on the other, the utmost accuracy of the emitter position was not required to obtain a good signal intensity.

**Figure 3.**
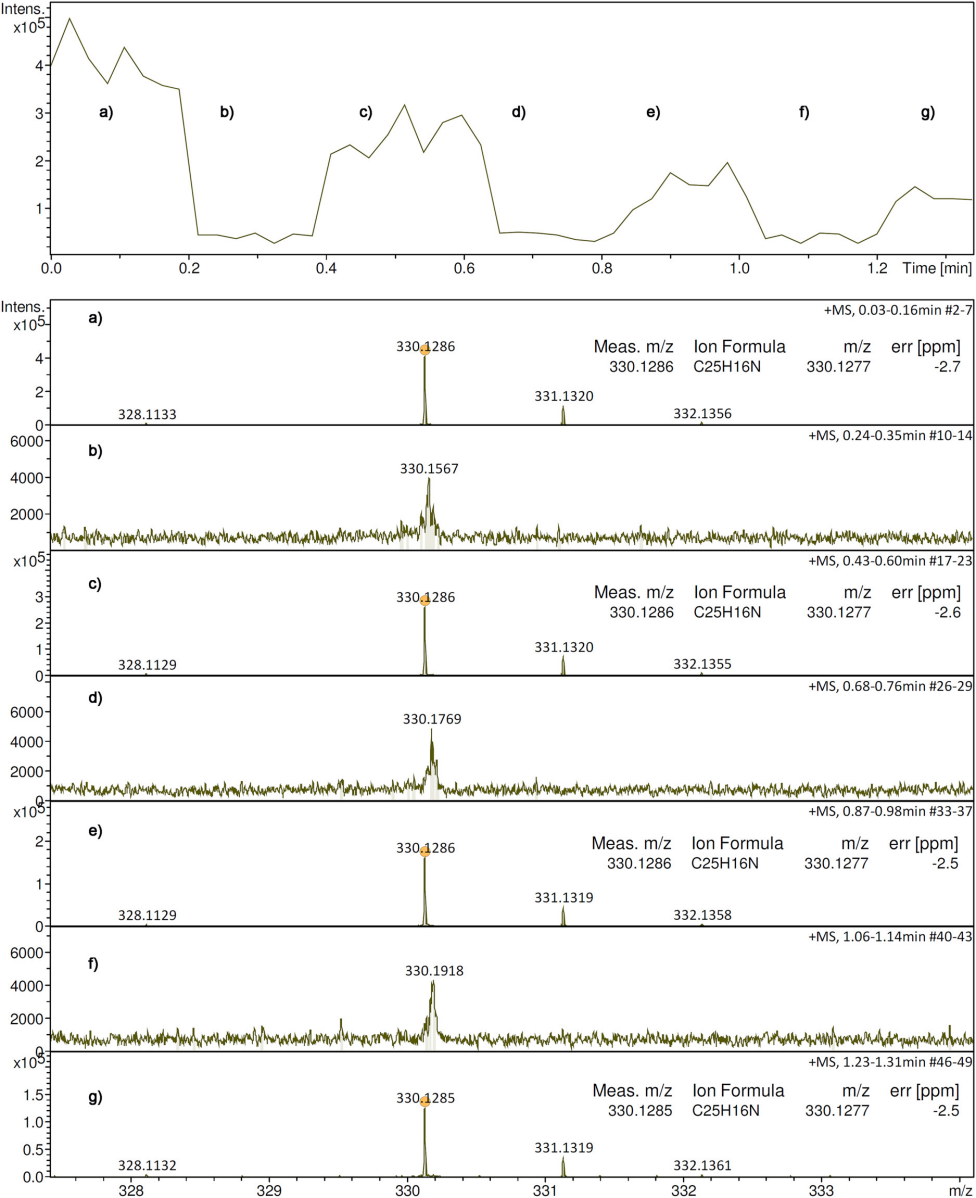
Some cycles of retracting and repositioning the emitter during an elongated run. The top part shows the base peak chromatogram (BPC) with labels marking the sections. Sections **a)**, **c)**, **e)**, and **g)** of the TIC correspond to acquisitions with the emitter in the correct position, the gaps where the intensity drops indicate the retraction of the emitter. Numbers of the respective transients accumulated and corresponding time intervals are annotated to the spectra. The sharp signal at *m/z* 330.1285 corresponds to the [M + H]^+^ ion of 1-aza-[6]helicene whereas the wide bump in the base lines of **b)**, **d)** and **f)** is due to electronic noise.

The recovery of the signal across some cycles of retracting the emitter by several cm and then repositioning it at the spray shield was tested as another criterion of robustness of operation. This test was done using the [M + H]^+^ ion of 1-aza-[6]helicene, *m/z* 330.1286, during an elongated run. The spectra were acquired in chromatography mode and the base peak chromatogram (BPC) was observed in relationship to the emitter location. For higher temporal resolution, this set of spectra was acquired using short 256 k data point transients. The top part of Figure 3 shows the BPC with labels marking the sections. Sections **a)**, **c)**, **e)**, and **g)** of the BPC correspond to acquisitions with the emitter in the correct position, the gaps where the intensity drops, indicate the retraction of the emitter. While the intensity of the peak at *m/z* 330.1286 immediately recovered when the emitter returned at the spray shield and resumed desorption/ionization, it slowly decreased during the 1.4-min long run as the sample was depleted toward the end of the period. It should be noted, that the mass accuracy was not affected by the interruptions but stayed at 2.5–2.7 ppm in all four APFD spectra. Even though the high voltage was constantly supplied, no discharges occurred when the emitter returned to the spray shield electrode.

### Reproduction of APFD spectra

The above two samples were selected for testing basic hardware properties because their behavior in APFD was known from previous work.^46^ In fact, the spectra obtained with the new setup were identical to those generated with the earlier setup based on a nanoESI source. Nonetheless, to be sure, some more compounds the APFD spectra of which were already known were revisited in order to corroborate the basic performance of the new assembly. In addition to the desorption of preformed positive ions of polar and ionic compounds the new assembly should also yield molecular ions of polycyclic aromatic hydrocarbons^47^ and allow for the application in negative-ion mode, e.g., for surfactants in commercial detergents.^48^ In other words, an at least equivalent performance should be guaranteed with the new APFD setup.

Thus, the APFD spectra of the basic polypropylene glycol oligomer Jeffamine M-2005, of the highly basic polycyclic aromatic 1-aza-[6]helicene, of benzo[a]pyrene, and of 1,1,4,4-tetraphenylbutadiene were acquired to test positive-ion performance while a shower gel was run to check negative-ion APFD. In brief, the spectra were essentially identical to those described in aforementioned APFD studies (Supplemental Figures S10–S14). Most importantly, the new setup delivered molecular ions by field ionization equally well.

### Emitter heating and observation

**Figure 4.**
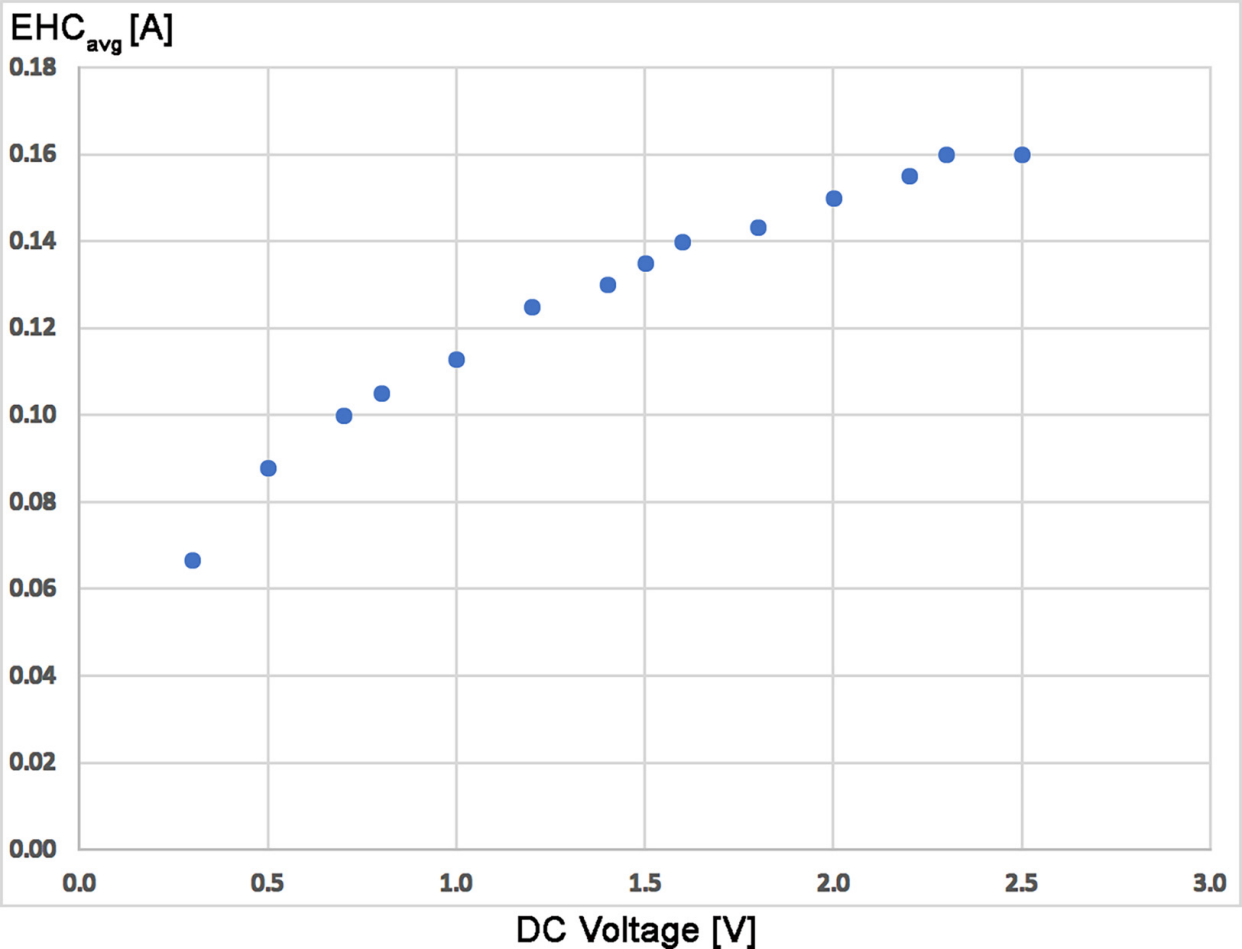
Heating characteristics of activated 13-µm emitters. The EHC is an average of seven runs using different emitters. Visible glow normally appeared at 1.8 V and 0.14 A, rupture tended to occur beyond 2.3 V and 0.16 A.

**Figure 5.**
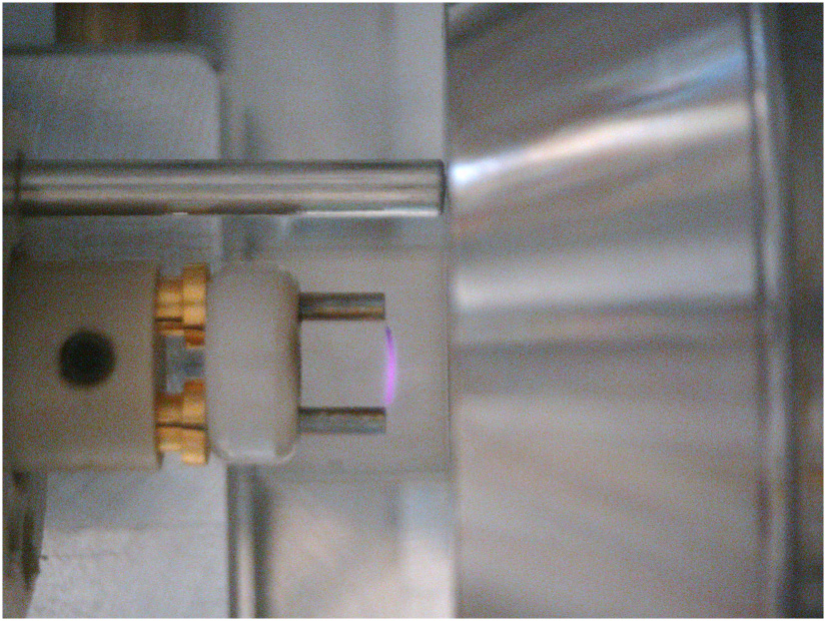
Screenshot by the USB microscope camera showing a glowing emitter close to the upper EHC limit at 2.2 V and 0.16 A. The pinkish color of the glow is caused by the USB camera while upon visual inspection it appears orange as expected.

While resistive heating of the emitter is common practice in FD and LIFDI, this option was missing in the initial setup for APFD. The application of an emitter heating current (EHC) induces some surface mobility of otherwise solid immobile analytes on the emitter, and thereby, expands the range of compounds FD or LIFDI may be applied to. Therefore, the pins of the emitter were connected to a DC power supply and the emitter was observed using the USB microscope camera while the voltage was ramped. A visible glow was observed from 1.8 V and 0.14 A onward until the emitter would shine bright and break at 2.3 V and 0.16 A. Emitter heating voltages and corresponding EHC were repeatedly determined using a set of emitters and were found to be reproducible within a narrow range, i.e., currents were reproducible within 0.01 A at any voltage (Figure 4). In routine operation this meant that a slow manual increase of the supply voltage to a maximum of 2.0 V would reliably cause to emitter to glow bright red yet without any loss in activity or even damage (Figure 5). The procedure of emitter heating is also documented in a video (Supplementary Material). Figure 5 and the video sequence also demonstrate the usefulness of the USB microscope camera mounted onto the sliding stage. The correct color of the glowing emitter can be seen in Supplemental Figure S15. When comparing the EHCs in vacuum FD or LIFDI to those in APFD, comparable glowing of the emitter appeared at 0.12 A in vacuum while it required 0.16 A at the open atmosphere. This increase in EHC was attributed to convective heat transport from the emitter into the environment.

### Advancements by applying an EHC

Employing an EHC ramp meant that it would be advantageous for spectral acquisition to proceed in a time-resolved mode. Thus, all following spectra were acquired using the instrument's chromatography mode where each transient was filed separately.

**Figure 6.**
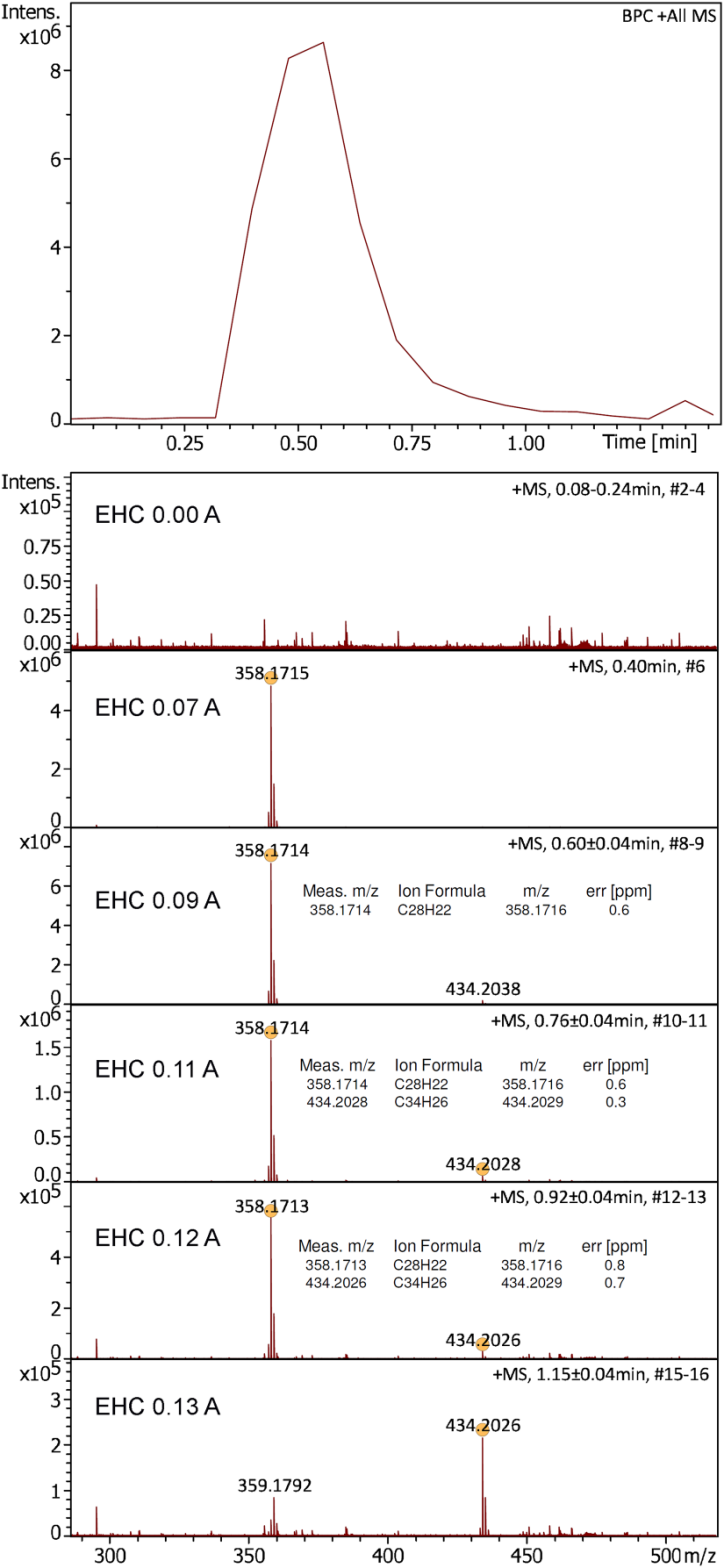
Positive-ion APFD spectra of 1,1,4,4-tetraphenylbutadiene as acquired while the EHC was raised manually from 0.00 A at 0.0 V to 0.13 A at 1.5 V. The onset of desorption/ionization at 0.07 A is clearly reflected in the base peak chromatogram (*top*). APFD settings: ion accumulation 2.0 s per transient, shield at −4.5 kV, cap at −5.5 kV, dry gas at 1.5 l min^–1^ and 140 °C.

While the APFD spectrum of a sample was being acquired, the EHC was slowly raised under manual control up to a level where the signal had ceased after sample had been consumed. In case of the positive-ion APFD spectrum of 1,1,4,4-tetraphenylbutadiene the EHC was ramped up to 0.13 A at 1.5 V during acquisition. The effect of using an EHC became immediately obvious as the onset of strong desorption/ionization occurred at an EHC of about 0.07 A and continued until the sample was consumed (Figure 6). Initially, the molecular ion of 1,1,4,4-tetraphenylbutadiene, [C_28_H_22_]^+•^, *m/z* 358.1715, appeared as the only signal. Later, when the EHC reached 0.10 A, an impurity, [C_34_H_26_]^+•^, *m/z* 434.2026, was detected in addition. This partial fractionation of the sample corresponded to what happens in vacuum FD when the EHC is being ramped.^49–51^ A repetition of this experiment and the application of an EHC ramp to the APFD analysis of Jeffamine M-2005 confirmed the usefulness of emitter heating in APFD as well as a behavior analogous to what has just been described (Supplemental Figures S16–S17).

**Figure 7.**
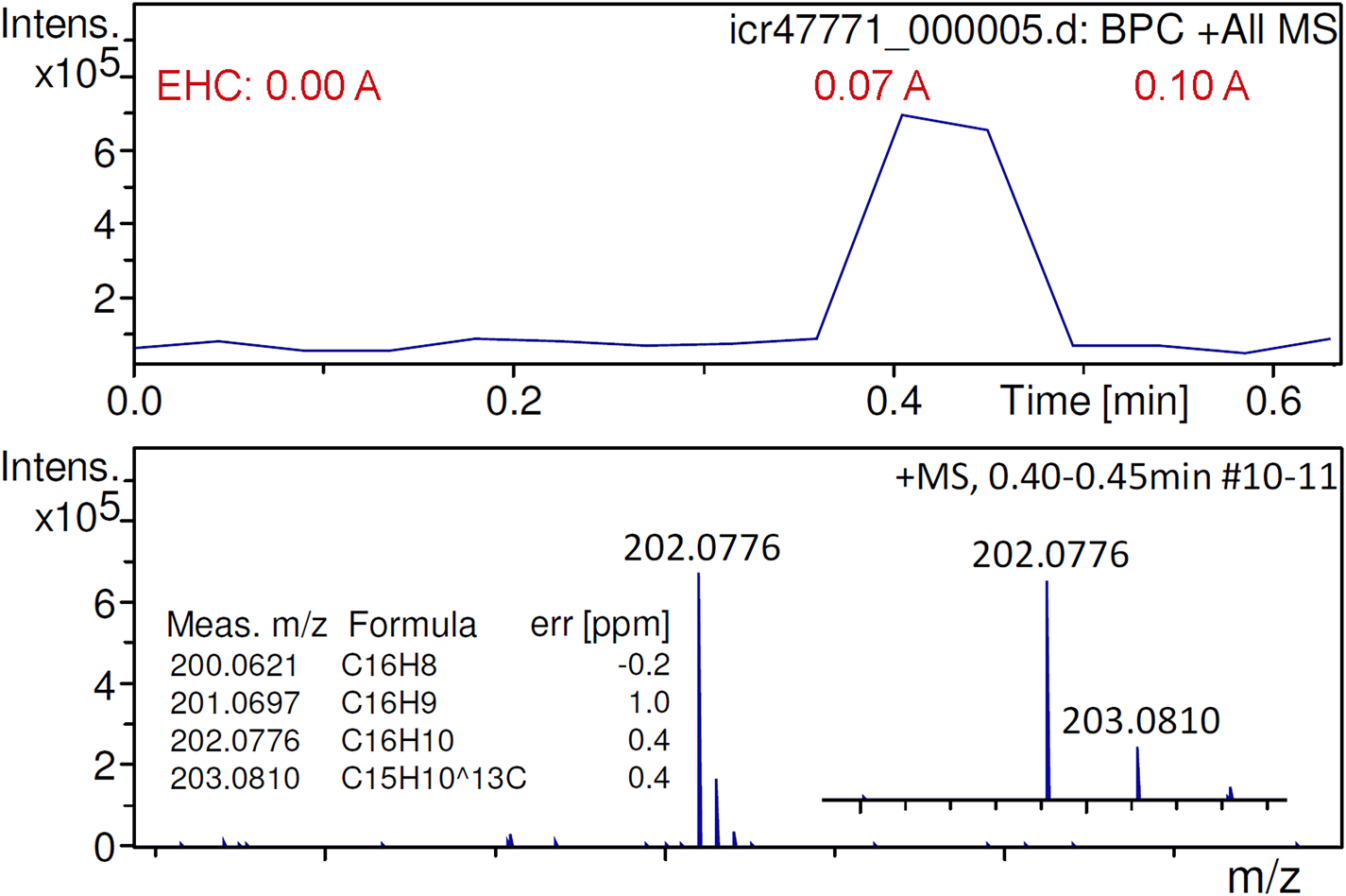
Positive-ion APFD spectrum of fluoranthene showing the molecular ion peak at *m/z* 202.0776. Two transients (#10–11) were summarized while the EHC was raised manually from 0.00 A at 0.0 V to 0.10 A at 0.7 V. The onset of desorption/ionization at about 0.07 A can be seen in the base peak chromatogram. APFD settings: ion accumulation 1.0 s per transient, shield at −4.7 kV, cap at −5.5 kV, dry gas at 1.2 l min^–1^ and 60 °C.

The molecular ion of fluoranthene, [C_16_H_10_]^+•^, had been difficult to generate with the initial APFD setup and had only been observed in the presence of residual benzo[a]pyrene.^47^ The present setup allowed quick and effective desorption/ionization to be achieved, which led to an improved molecular ion peak intensity at *m/z* 202.0776 (calc. 202.0777) (Figure 7). The signal would normally appear when the EHC reached 0.07 A and last for two or three transients yielding a peak due to [C_16_H_10_]^+•^ at an intensity of about 7 × 10^5^ counts. The use of an EHC permitted to decouple the event of desorption from the desolvation gas temperature that could now be set as low as 60 °C, which avoided losses of the sample by evaporation prior to the start of the acquisition. This way, the use of an EHC also contributed to an improved reproducibility of the acquisition of APFD spectra of fluoranthene.

**Figure 8.**
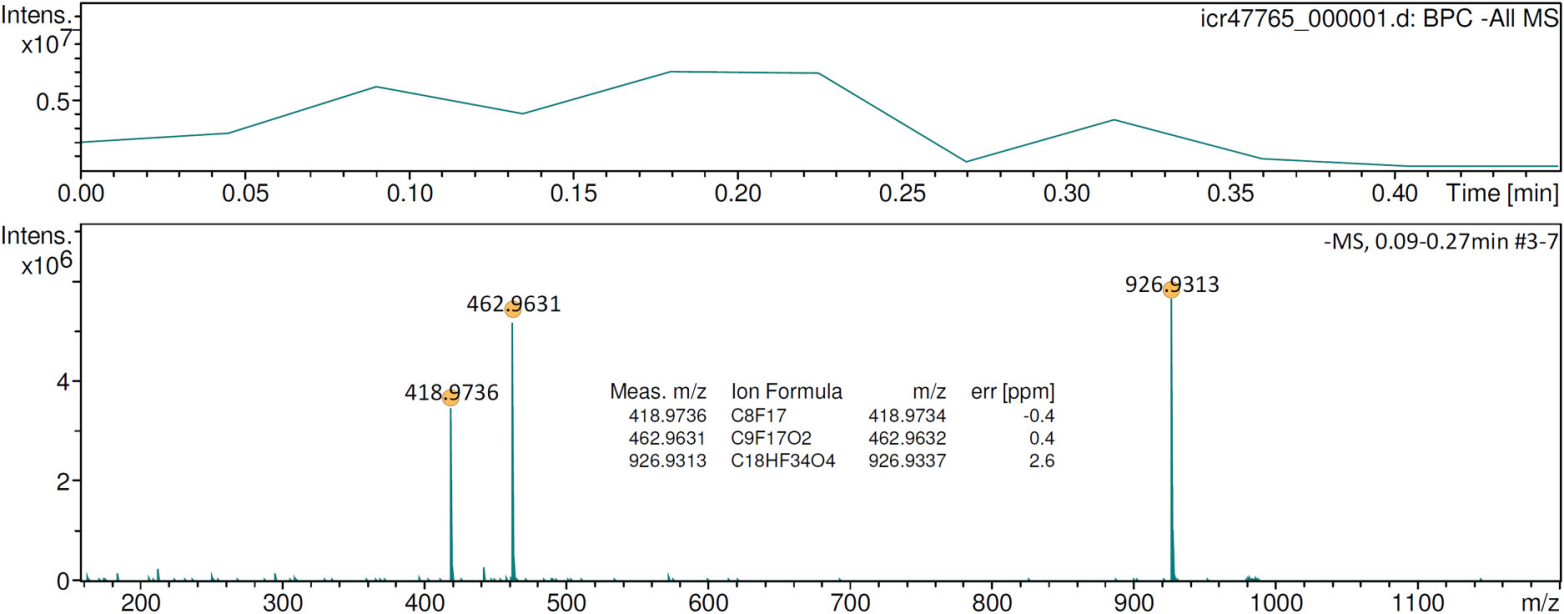
Negative-ion APFD spectrum of ca. 0.2 µg perfluorononanoic acid (PFNA) acquired while raising the EHC to 0.12 mA. The spectrum shown was the sum of the transients #3–7 and shows the [M–H]^–^ ion, [C_9_F_17_O_2_]^–^, *m/z* 462.9631, a fragment by loss of CO_2_, [C_8_F_17_]^–^, *m/z* 418.9736, and the [2M–H]^–^ cluster ion, [C_18_HF_34_O_4_]^–^, *m/z* 926.9313. APFD settings: ion accumulation 1.0 s per transient, shield at 4.2 kV, cap at 5.0 kV, dry gas at 1.2 l min^–1^ and 140 °C.

Perfluorononanoic acid (PFNA) is solid at room temperature and could previously only be analyzed by negative-ion APFD when it was applied to the emitter in a matrix of glycerol.^46^ Otherwise, the surface mobility of PFNA would not have been sufficient to allow for any desorption/ionization. With emitter heating in operation, neat PFNA was sufficiently mobilized on the emitter surface to start desorption ionization of the [M–H]^–^ ion as soon as the EHC reached about 0.05 A (Figure 8). Depending on the actual heating rate, either the [M–H]^–^ ion, [C_9_F_17_O_2_]^–^, *m/z* 462.9631, plus the fragment by loss of CO_2_, [C_8_F_17_]^–^, *m/z* 418.9736, or the [2M–H]^–^ cluster ion, [C_18_HF_34_O_4_]^–^, *m/z* 926.9313, were dominating the spectrum. Here, in contrast to the situation without an EHC, the glycerol matrix was not needed anymore to acquire the APFD spectrum of PFNA. Still, APFD was soft enough to generally favor the formation of the [2M–H]^–^ cluster ion over the [M–H]^–^ ion.

**Figure 9.**
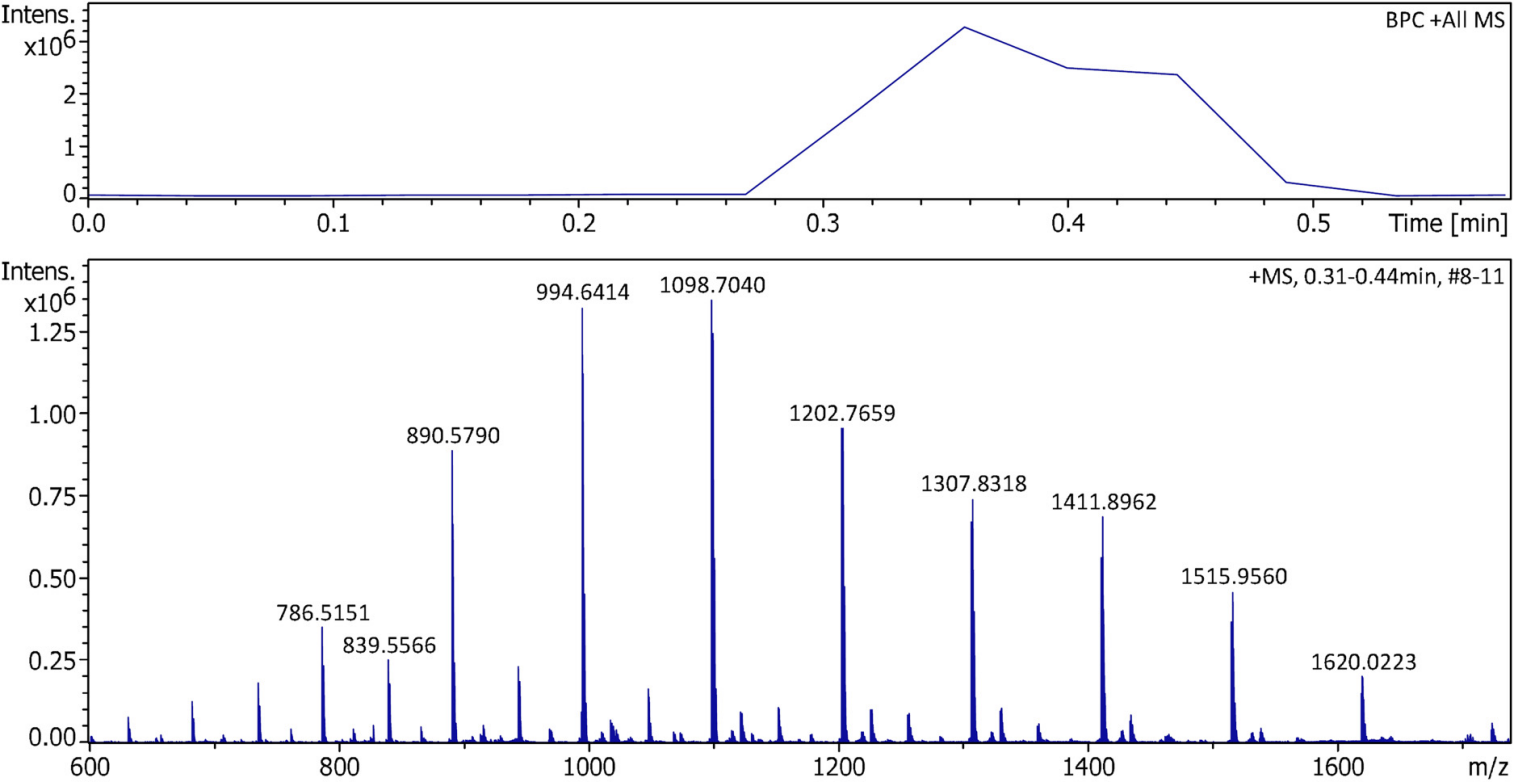
Positive-ion APFD spectrum of 3.5 µg of polystyrene of average molecular weight of 1 ku obtained using an EHC ramp up to 0.15 A. The spectrum shown was the sum of the transients #8–11. APFD settings: ion accumulation 1.0 s per transient, shield at −4.8 kV, cap at −5.5 kV, dry gas at 1.5 l min^–1^ and 140 °C. The series of molecular ions of the general formula [C_4_H_10_(C_8_H_8_)_n_]^+•^ started to appear at ca. 0.09 A and vanished at 0.13 A after the sample had been consumed. Molecular ions were detected from the 6mer (*m/z* 682) to the 16mer (*m/z* 1724).

The EHC also enabled the APFD analysis of polystyrene (PS), which had not been possible with the initial setup. When butyl-terminated PS of average molecular weight of either 560 u or 1 ku was subjected to positive-ion APFD analysis, the onset of desorption only occurred after some heating was applied. The dominant series of ions corresponded to molecular ions of the general formula [C_4_H_10_(C_8_H_8_)_n_]^+•^, at even *m/z* values, mostly ions from *n* = 6–11 in case of PS 560 and from *n* = 6–16 in case of PS 1k (Figure 9). While in LIFDI, similar PS samples were not found to undergo notable fragmentations,^29^ in APFD, some fragment ions occurred in addition to the molecular ions. To these fragment ions compositions with either C_2_H^•^ or C_4_H_3_^•^ less than the next higher molecular ions were assigned (Supplemental Figures S18–S19).

### Transfer to the timsTOFflex instrument

For compatibility testing, the APFD source assembly was mounted to the timsTOFflex instrument as described (Supplemental Figs. S5–A8) and spectra were acquired using source settings analogous to those reported for the FT-ICR mass spectrometer. The source worked immediately and did not have any compatibility issues. In positive ion mode, the spectra of 1,1,4,4-tetraphenylbutadiene, of fluoranthene, and of PS 1k were recorded, and for the most part, were found to exhibit the same appearance as reported above. These three spectra are provided in the Supplementary Materials section (Supplemental Figs. S20–S22). Among these, the base peak chromatogram and the spectrum of 1,1,4,4-tetraphenylbutadiene had the closest resemblance to those from FT-ICR.

The spectrum of fluoranthene was clearly better in terms of signal-to-noise ratio and the duration the instrument was capable of recording a useful signal. It is noteworthy that the timsTOFflex instrument already detected the molecular ion from the beginning, i.e., without or very low EHC at an intensity of about 3 × 10^4^ counts while the signal reached an almost two orders of magnitude higher level of 4 × 10^6^ counts during the most active desorption period. This is where the low-mass cut-off at around *m/z* 170 of the ApexQe mass spectrometer already had an adverse effect on ion transmission whereas the timsTOFflex played at its strengths.

In case of PS 1k, there were minor differences, for example in molecular weight distributions, that might not necessarily have been related to the APFD process itself, but could also have been caused by somewhat different ion transfer parameters between the two instruments.

**Figure 10.**
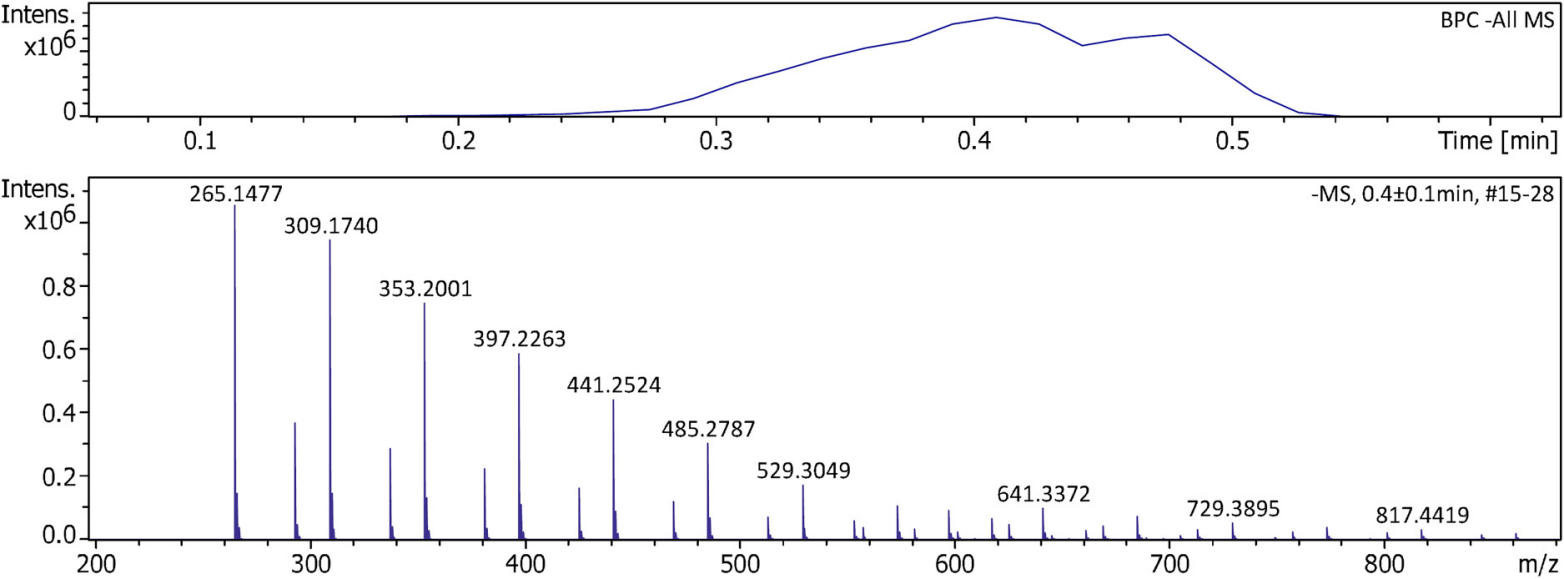
Negative-ion APFD spectrum of shower gel acquired using the timsTOFflex instrument. APFD settings: ion accumulation 1.0 s per spectrum, shield at 4.3 kV, dry gas at 4.0 l min^–1^ and 150 °C, and EHC ramp up to 0.14 A.

Negative-ion APFD was finally tested on the timsTOFflex using PFNA and the shower gel. Unsurprisingly, there were also no major differences to be observed. The shower gel desorbed upon gentle heating of the emitter and exhibited the series of organic sulfate ions already known (Figure 10).^48^ For more detail and formula assignments to the main signals see Supplemental Figure S23.

The spectrum of PFNA also showed the [M–H]^–^ ion, [C_9_F_17_O_2_]^–^, *m/z* 462.9627, a fragment by loss of CO_2_, [C_8_F_17_]^–^, *m/z* 418.9728, and the [2M–H]^–^ cluster ion, [C_18_HF_34_O_4_]^–^, *m/z* 926.9324, as the base peak (Supplemental Figure S24).

## Conclusion

The new APFD source assembly enabled a notable advancement of the method in terms of robustness and reliability of operation. In addition, it introduced means to apply an emitter heating current and to observe the emitter during operation. While comparatively simple in its construction, it permitted much quicker and easier operation than the previous explorative setup. Moreover, the implementation of an EHC allowed to expand the accessible range of samples and to omit glycerol matrix in case of PFNA. The use of an EHC was particularly beneficial when combined with the instrument's chromatography mode that delivered data allowing to extract only the relevant portion of the TIC with good analyte signal to create the final APFD spectrum. Furthermore, the improved and variable source mechanics permitted the use of the device in combination with different mass spectrometers as exemplified here by the combination with both the ApexQe and the timsTOFflex instruments. The adaptation to the modern instrumental platform alone allowed for a higher speed of spectral acquisition and better sensitivity and yielded easier access to the low *m/z* range (Table 2). So far, the results encourage further investigations of both fundamentals and applications of APFD.

**Table 2.** Comparison between previous and new emitter assembly for APFD.

Previous setup	New assembly	Effect of changes
Based on Bruker nanoESI source for Apex series of instruments with hinges on the right side	Custom-built frame with adjustable slide-in mount for emitter holder	Robust mount allowing for quick and reproducible exchange of ion sources
Restricted to use with Bruker Apex series of instruments	Hinges and holders for the clip can be swapped to either side of the frame	Versatile source assembly for APFD that fits all current Bruker instruments with AP interface
Restricted to > *m/z* 170 range of Bruker ApexQe instruments	Can be used in any *m/z* range accessible by a suitable instrument, typically *m/z* 50–5000	Expanded *m/z* range, especially to the low-mass side
Emitter mounted to slot of clamp and screwed into holder of nanoESI source	Emitter slides into sockets of a probe tip	Emitter easier and quicker to mount and improved positioning
Emitter orientation roughly vertical	Emitter orientation is horizontal	Sample solution can even be transferred onto emitter while mounted
Emitter orientation at an angle to the surface of the counter electrode	Emitter orientation along its full lengths always parallel to counter electrode	Symmetric extraction field for APFD and less risk of discharges
Emitter orientation *x*,*y*,*z*-variable with respect to entrance of spray shield, and thus, counter electrode	Still *x*,*y*-shiftable with respect to entrance of spray shield for fine adjustment after mounting the source	Emitter orientation reproducible after being fixed once and always parallel to counter electrode
Observation via CCD camera at 45° sideways from top	Observation via USB microscope vertically from top	Less hardware and parallax-free observation
Analog video output to small standalone monochrome monitor	Color video signal displayed in a window on computer screen	Capability to document emitter site via screenshots and video clips
Probe with no electrical supplies, just grounded	Probe tip grounded and provides sockets connected to emitter heating power supply	Enables emitter heating current (EHC) to be applied in APFD
Emitter temperature roughly determined by desolvation gas alone	Emitter temperature determined by desolvation gas and EHC if intended	EHC helps to expand the mass range and range of analytes and allows to speed up the analyses

## Supplemental Material

sj-pdf-1-ems-10.1177_14690667241236073 - Supplemental material for Robust and versatile assembly for emitter positioning, observation, and heating in atmospheric pressure field desorption mass spectrometry Supplemental material, sj-pdf-1-ems-10.1177_14690667241236073 for Robust and versatile assembly for emitter positioning, observation, and heating in atmospheric pressure field desorption mass spectrometry by Jan Schweinfurth, H. Bernhard Linden and Jürgen H. Gross in European Journal of Mass Spectrometry

**Figure:** Video 1.

## References

[1] Beckey HD . Field-Ionization Mass Spectrometry. Elmsford: Pergamon, 1971.

[2] Beckey HD . Principles of Field Desorption and Field Ionization Mass Spectrometry. Oxford: Pergamon Press, 1977.

[3] Prókai L . Field Desorption Mass Spectrometry. New York: Marcel Dekker, 1990.

[4] Gross JH . From the discovery of field ionization to field desorption and liquid injection field desorption/ionization-mass spectrometry—A journey from principles and applications to a glimpse into the future. Eur J Mass Spectrom 2020; 26: 241–273. 10.1177/1469066720939399 PMC7383431 32605392

[5] Chen LC Rahman MM Hiraoka K . Non-vacuum field desorption ion source implemented under super-atmospheric pressure. J Mass Spectrom 2012; 47: 1083–1089. 22899518 10.1002/jms.3062

[6] Chen X Cooks RG . Accelerated reactions in field desorption mass spectrometry. J Mass Spectrom 2018; 53: 942–946. 29935122 10.1002/jms.4254

[7] Heinen HJ Giessmann U Röllgen FW . Field desorption of electrolytic solutions using untreated wire emitters. Org Mass Spectrom 1977; 12: 710–715.

[8] Veith HJ . Alkali ion addition in FD mass spectrometry. Cationization and protonation-ionization methods in the application of nonactivated emitters. Tetrahedron 1977; 33: 2825–2828.

[9] Röllgen FW Giessmann U Borchers F , et al. Collisional activation spectra of [M + Li]^+^, [M + Na]^+^ and [M + K]^+^ ions formed by field desorption of some monosaccharides. Org Mass Spectrom 1978; 13: 459–461.

[10] Keough T DeStefano AJ . Acid-enhanced field desorption mass spectrometry of zwitterions. Anal Chem 1981; 53: 25–29.

[11] Davis SC Neumann GM Derrick PJ . Field desorption mass spectrometry with suppression of the high field. Anal Chem 1987; 59: 1360–1362.

[12] Beckey HD Hilt E Schulten H-R . High temperature activation of emitters for field ionization and field desorption spectrometry. J Phys E: Sci Instrum 1973; 6: 1043–1044.

[13] Linden HB Hilt E Beckey HD . High-rate growth of dendrites on thin wire anodes for field desorption mass spectrometry. J Phys E: Sci Instrum 1978; 11: 1033–1036.

[14] Rabrenovic M Ast T Kramer V . Alternative organic substances for generation of carbon emitters for field desorption mass spectrometry. Int J Mass Spectrom Ion Phys 1981; 37: 297–307.

[15] Linden HB . Liquid injection field desorption ionization: a new tool for soft ionization of samples including air-sensitive catalysts and non-polar hydrocarbons. Eur J Mass Spectrom 2004; 10: 459–468. 10.1255/ejms.655 15302970

[16] Gross JH Nieth N Linden HB , et al. Liquid injection field desorption/ionization of reactive transition metal complexes. Anal Bioanal Chem 2006; 386: 52–58. 16773301 10.1007/s00216-006-0524-0

[17] Muhr M Heiß P Schütz M , et al. Enabling LIFDI-MS measurements of highly air sensitive organometallic compounds: a combined MS/glovebox technique. Dalton Trans 2021; 50: 9031–9036. 33970171 10.1039/d1dt00978h

[18] Taubert J Vogt M Langer R . Mass spectrometric detection of ion pairs containing rigid copper clusters and weakly coordinating counter ions using liquid injection field desorption/ionisation. Eur J Mass Spectrom 2023; 29: 68–74. 10.1177/14690667221139419 36437773

[19] Linden MH Linden HB . Unprecedented intact radical anions, closed shell anions, cluster ions, and traditional cations and radical cations by LIFDI–MS. Eur J Mass Spectrom 2023; 29: 5–11. 10.1177/14690667221146079 36605010

[20] Schaub TM Hendrickson CL Qian K , et al. High-Resolution field desorption/ionization Fourier transform ion cyclotron resonance mass analysis of nonpolar molecules. Anal Chem 2003; 75: 2172–2176. 12720358 10.1021/ac020627v

[21] Marshall AG Rodgers RP . Petroleomics: the next grand challenge for chemical analysis. Acc Chem Res 2004; 37: 53–59. 14730994 10.1021/ar020177t

[22] Schaub TM Linden HB Hendrickson CL , et al. Continuous-flow sample introduction for field desorption/ionization mass spectrometry. Rapid Commun Mass Spectrom 2004; 18: 1641–1644.

[23] Linden HB Gross JH . A liquid injection field desorption/ionization-electrospray ionization combination source for a Fourier transform ion cyclotron resonance mass spectrometer. J Am Soc Mass Spectrom 2011; 22: 2137–2144. 22006404 10.1007/s13361-011-0259-9

[24] Linden HB Gross JH . Reduced fragmentation in liquid injection field desorption/ionization-Fourier transform ion cyclotron resonance mass spectrometry by use of helium for the thermalization of molecular ions. Rapid Commun Mass Spectrom 2012; 26: 336–344. 22223321 10.1002/rcm.5335

[25] Miyamoto K Fujimaki S Ueda Y . Development of a new electron ionization/field ionization ion source for gas chromatography/time-of-flight mass spectrometry. Rapid Commun Mass Spectrom 2009; 23: 3350–3354. 19764073 10.1002/rcm.4256

[26] Breunig HJ Linden HB Moldovan O . Liquid injection field desorption ionization mass spectrometry of cyclic metal carbonyl complexes with tetra-antimony ligands. J Am Soc Mass Spectrom 2013; 24: 164–166. 23250666 10.1007/s13361-012-0522-8

[27] Genuit W Chaabani H . Comprehensive two-dimensional gas chromatography-field ionization time-of-flight mass spectrometry (GCxGC-FI-TOFMS) for detailed hydrocarbon middle distillate analysis. Int J Mass Spectrom 2017; 413: 27–32.

[28] Giri A Coutriade M Racaud A , et al. Compositional elucidation of heavy petroleum base oil by GC × GC-EI/PI/CI/FI-TOFMS. J Mass Spectrom 2019; 54: 148–157. 30536759 10.1002/jms.4319

[29] Linden MH Linden HB Nieth N , et al. Self-Supplied liquid injection field desorption/ionization ion source for an orthogonal time-of-flight instrument. J Am Soc Mass Spectrom 2019; 30: 2358–2368. 31376121 10.1007/s13361-019-02297-1

[30] Linden MH Linden HB Gross JH . Negative-ion field desorption revitalized by using liquid injection field desorption/ionization-mass spectrometry on recent instrumentation. Anal Bioanal Chem 2021; 413: 6845–6855. 34494122 10.1007/s00216-021-03641-9 PMC8551092

[31] Cooks RG Ouyang Z Takats Z , et al. Ambient mass spectrometry. Science 2006; 311: 1566–1570. 16543450 10.1126/science.1119426

[32] Venter A Nefliu M Cooks RG . Ambient desorption ionization mass spectrometry. Trends Anal Chem 2008; 27: 284–290.

[33] Feider CL Krieger A DeHoog RJ , et al. Ambient ionization mass spectrometry: recent developments and applications. Anal Chem 2019; 91: 4266–4290. 30790515 10.1021/acs.analchem.9b00807 PMC7444024

[34] Hiraoka K Nishidate K Mori K , et al. Development of probe electrospray using a solid needle. Rapid Commun Mass Spectrom 2007; 21: 3139–3144. 17708527 10.1002/rcm.3201

[35] Yang Y Deng J Yao Z-P . Field-induced wooden-tip electrospray ionization mass spectrometry for high-throughput analysis of herbal medicines. Anal Chim Acta 2015; 887: 127–137. 26320794 10.1016/j.aca.2015.06.025

[36] Wu M-L Chen T-Y Chen Y-C , et al. Carbon fiber ionization mass spectrometry for the analysis of analytes in vapor, liquid, and solid phases. Anal Chem 2017; 89: 13458–13465. 29155550 10.1021/acs.analchem.7b03736

[37] Selvaprakash K Chen Y-C . Using an insulating fiber as the sampling probe and ionization substrate for ambient ionization–mass spectrometric analysis of volatile, semi-volatile, and polar analytes. Anal Bioanal Chem 2022; 414: 4633–4643. 35445835 10.1007/s00216-022-04080-w

[38] Kuo C-P Shiea J . Application of direct electrospray probe to analyze biological compounds and to couple to solid-phase microextraction to detect trace surfactants in aqueous solution. Anal Chem 1999; 71: 4413–4417. 21662868 10.1021/ac990049r

[39] Hong C-M Lee C-T Lee Y-M , et al. Generating electrospray from solutions predeposited on a copper wire. Rapid Commun Mass Spectrom 1999; 13: 21–25.

[40] Kuo C-P Yuan C-H Shiea J . Generation of electrospray from a solution predeposited on optical fibers coiled with a platinum wire. J Am Soc Mass Spectrom 2000; 11: 464–467. 10790851 10.1016/S1044-0305(00)00111-2

[41] Jeng J Shiea J . Electrospray ionization from a droplet deposited on a surface-modified glass rod. Rapid Commun Mass Spectrom 2003; 17: 1709–1713. 12872275 10.1002/rcm.1109

[42] Jeng J Lin C-H Shiea J . Electrospray from nanostructured tungsten oxide surfaces with ultralow sample volume. Anal Chem 2005; 77: 8170–8173. 16351172 10.1021/ac0512960

[43] Pirkl A Dreisewerd K Yew JY , et al. Field-based ion generation from microscale emitters on natural and artificial objects for atmospheric pressure mass spectrometry. Anal Bioanal Chem 2010; 397: 3311–3316. 19838683 10.1007/s00216-009-3184-z

[44] Moini M . Ultramark 1621 as a calibration/reference compound for mass spectrometry. II. Positive- and negative-ion electrospray ionization. Rapid Commun Mass Spectrom 1994; 8: 711–714.

[45] Flanagan JM. *Mass spectrometry calibration using homogeneously substituted fluorinated triazatriphosphorines*. Patent 5872357, US, 1997.

[46] Gross JH . Desorption of positive and negative ions from activated field emitters at atmospheric pressure. Eur J Mass Spectrom 2023; 29: 21–32. 10.1177/14690667221133388 PMC9903004 36254584

[47] Hoyer M Gross JH . Molecular ion formation on activated field emitters in atmospheric pressure field desorption mass spectrometry. Anal Bioanal Chem 2023; 415: 2307–2315. 36961573 10.1007/s00216-023-04652-4 PMC10115680

[48] Gross JH . Application of atmospheric pressure field desorption for the analysis of anionic surfactants in commercial detergents. Anal Bioanal Chem 2023; 415: 6421–6430. 37644322 10.1007/s00216-023-04917-y PMC10567867

[49] Maine JW Soltmann B Holland JF , et al. Emitter current programmer for field desorption mass spectrometry. Anal Chem 1976; 48: 427–429.

[50] Beckey HD . Experimental techniques in field ionisation and field desorption mass spectrometry. J Phys E: Sci Instrum 1979; 12: 72–83.

[51] Fraley DF Pedersen LG Bursey MM . Resistive heating of emitter wires for field desorption and ionization: a theory. Int J Mass Spectrom Ion Phys 1982; 43: 99–129.

[52] Zhang D Wu L Koch KJ , et al. Arginine clusters generated by electrospray ionization and identified by tandem mass spectrometry. Eur Mass Spectrom 1999; 5: 353–361.

[53] Soga T Kakazu Y Robert M , et al. Qualitative and quantitative analysis of amino acids by capillary electrophoresis-electrospray ionization-tandem mass spectrometry. Electrophoresis 2004; 25: 1964–1972. 15237395 10.1002/elps.200305791

[54] Sládková K Houška J Havel J . Laser desorption ionization of red phosphorus clusters and their use for mass calibration in time-of-flight mass spectrometry. Rapid Commun Mass Spectrom 2009; 23: 3114–3118. 19714708 10.1002/rcm.4230

[55] Kolářová L Prokeš L Kučera L , et al. Clusters of monoisotopic elements for calibration in (TOF) mass spectrometry. J Am Soc Mass Spectrom 2017; 28: 419–427. 27995502 10.1007/s13361-016-1567-x

