# Supplementary material for: Robust and versatile assembly for emitter positioning, observation, and heating in atmospheric pressure field desorption mass spectrometry
Source: Eur J Mass Spectrom (Chichester). 2024 Mar 15;30(2):103–15. doi: 10.1177/14690667241236073 (PMC11063571; doi:10.1177/14690667241236073)
Supplement: sj-pdf-1-ems-10.1177_14690667241236073 - Supplemental material for Robust and versatile assembly for emitter positioning, observation, and heating in atmospheric pressure field desorption mass spectrometry [file sj-pdf-1-ems-10.1177_14690667241236073.pdf]

Robust and versatile assembly for emitter positioning, observation, and heating in atmospheric pressure field desorption mass spectrometry

Supplementary Data

Jan Schweinfurth [1)], H. Bernhard Linden [2)], Jürgen H. Gross [3)]

[1] Institute of Inorganic Chemistry, Heidelberg University, Im Neuenheimer Feld 270, 69120 Heidelberg, Germany.

[2] Linden CMS, Auf dem Berge 25, 28844 Weyhe, Germany

[3] Institute of Organic Chemistry, Heidelberg University, Im Neuenheimer Feld 270, 69120 Heidelberg, Germany.

* Send correspondence to Jürgen H. Gross

ORCID 0000-0003-0748-2535

email juergen.gross@oci.uni-heidelberg.de

phone +49/6221/54-8409

Fig. S1. Design drawing of the custom-built mechanics of the APFD source assembly. This view shows the source along the *z*-axis. The entrance of the interface would be on the right side, the probe mount slides on the rail from left to right. This and the following three drawings are shown in the configuration for attachment to the timsTOFflex and all other modern Bruker instruments with AP interface, i.e., hinges on the left side, holders for the source door clip on the right. All four design drawings are reproduced at the same scale.

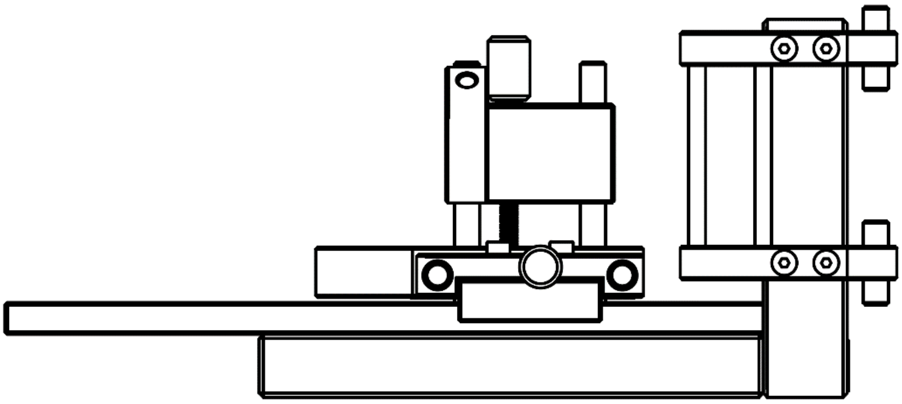

Fig. S2. Design drawing of the APFD source assembly showing the source in the *x*,*y*-plane looking along the *z*-axis when looking from the AP interface side. The bent clip in the middle takes up the probe tip.

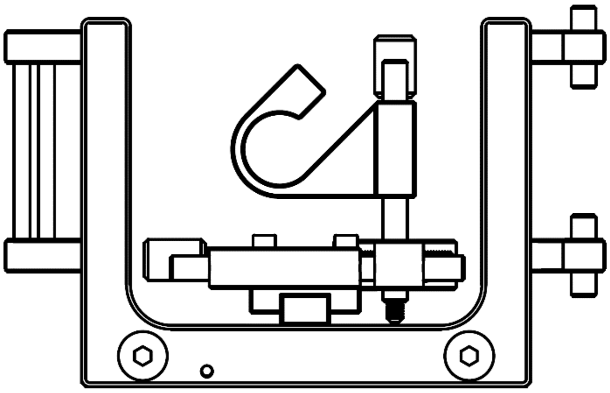

Fig. S3. Design drawing of the APFD source assembly from top, i.e., along the *y*-axis. The entrance of the interface would be on the bottom. The USB microscope stand fits into the round opening on the upper right side of the slider on the rail.

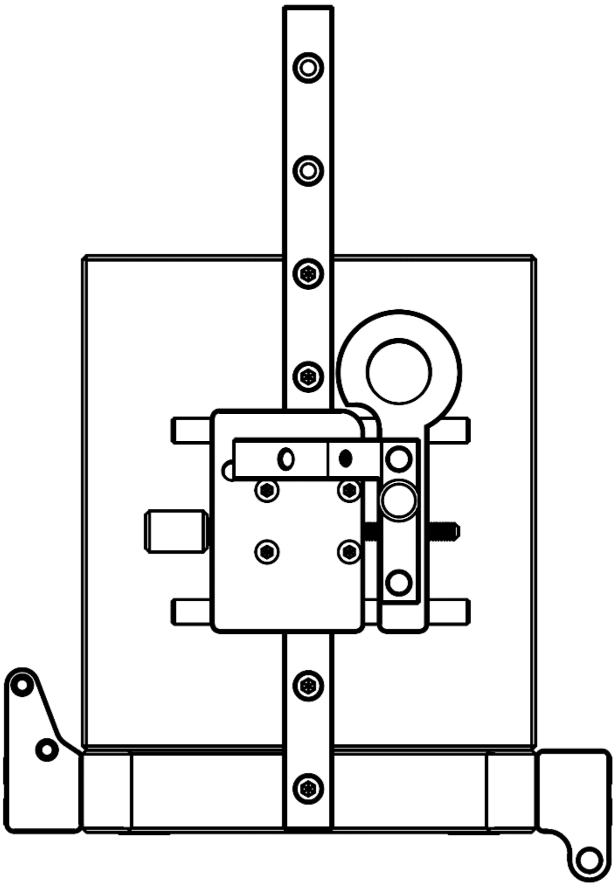

Fig. S4. Design drawing of the APFD source assembly in a 3D perspective.

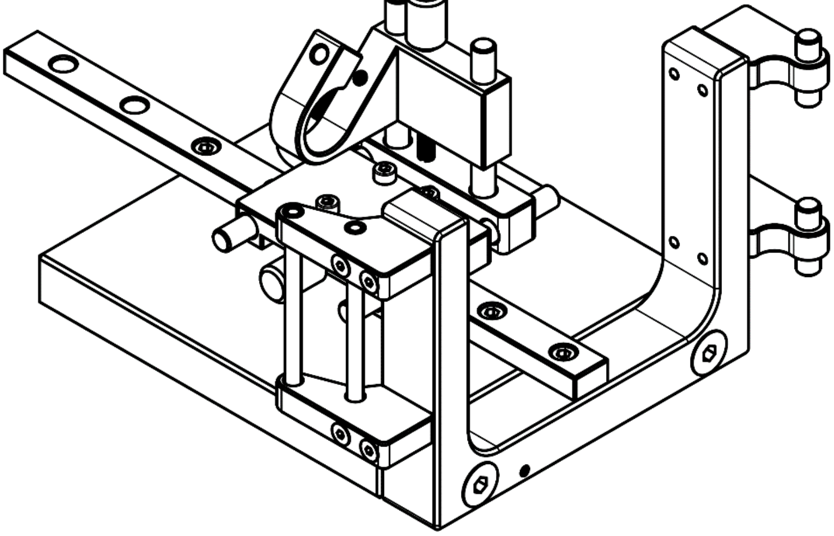

Figs. S5–S8. Photograph of the APFD source mounted to the Bruker timsTOFflex instrument (*top*). This configuration is the one that is compatible with all current Bruker instruments that are equipped with an AP interface. The conversion from the older ApexQe interface (*bottom left*) to modern Bruker AP interfaces with hinges on the left side (*bottom right*) is shown in a series of three photographs below as seen from the AP interface side. Swapping is done within minutes by simply loosening and tightening eight screws.

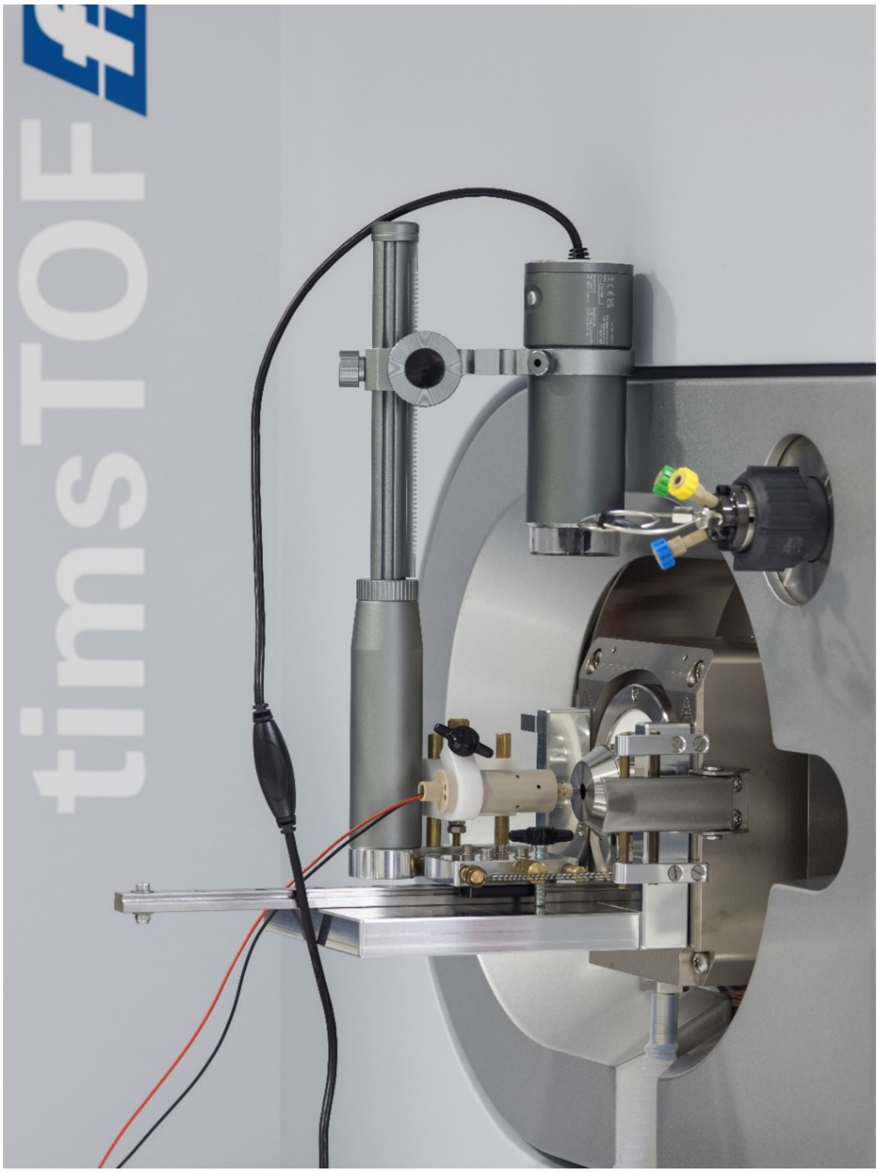

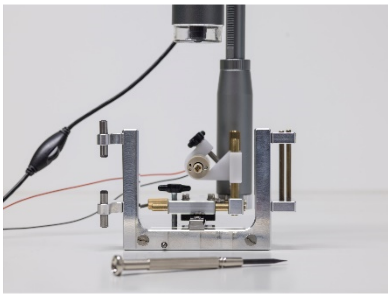	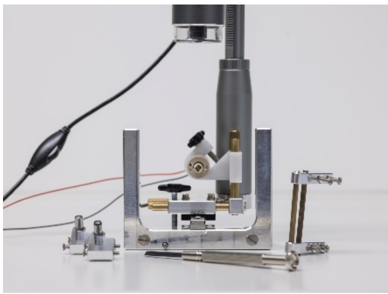	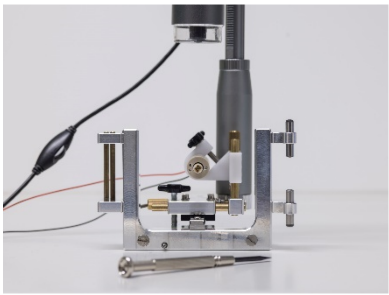
For ApexQe AP interface	“Transition state”	For current AP interfaces

Fig. S9. Effect of shifting the emitter sideways by 2 mm out of the central axis of the AP interface. Two runs with repeated location of the emitter at any of these positions were conducted and the data is summarized below. The spectrum exemplifies icr47224 2nd shift left. The stronger drop to the right side most probably is an effect of parallax error as the 2 mm shift was estimated each time by simply looking at the actual emitter position.

	Center	Up	Down	Left	Right
	Intensities in kCounts				
**icr47224**	2100	600	500	460	1400
	14000	1900	13500	8000	310
	4300				
**icr47225**	2900	4000	440	1000	240
	2000				
	700				
**Average**	4333	2167	4813	3153	650

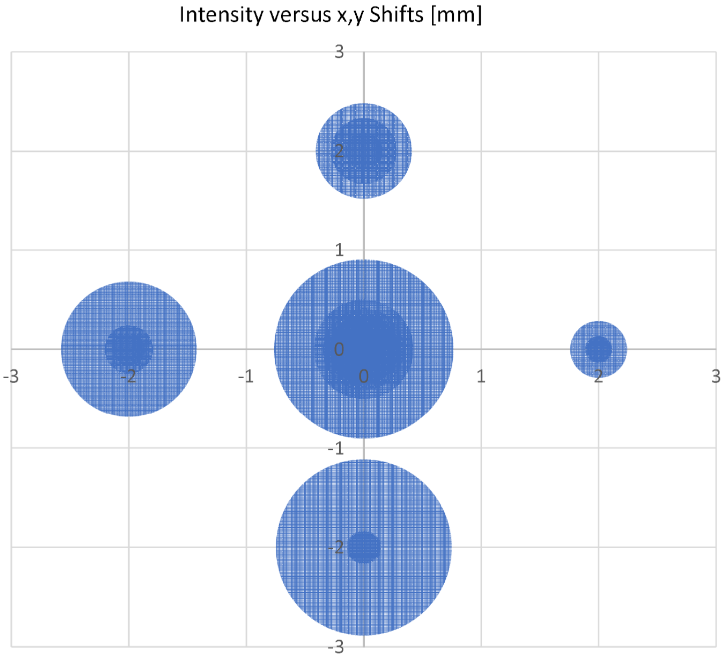

x Shift	y Shift	Intensity
0	0	2100
0	0	14000
0	0	4300
0	0	2900
0	0	2000
0	0	700
-2	0	460
-2	0	8000
-2	0	1000
2	0	1400
2	0	310
2	0	240
0	2	600
0	2	1900
0	2	4000
0	-2	500
0	-2	13500
0	-2	440

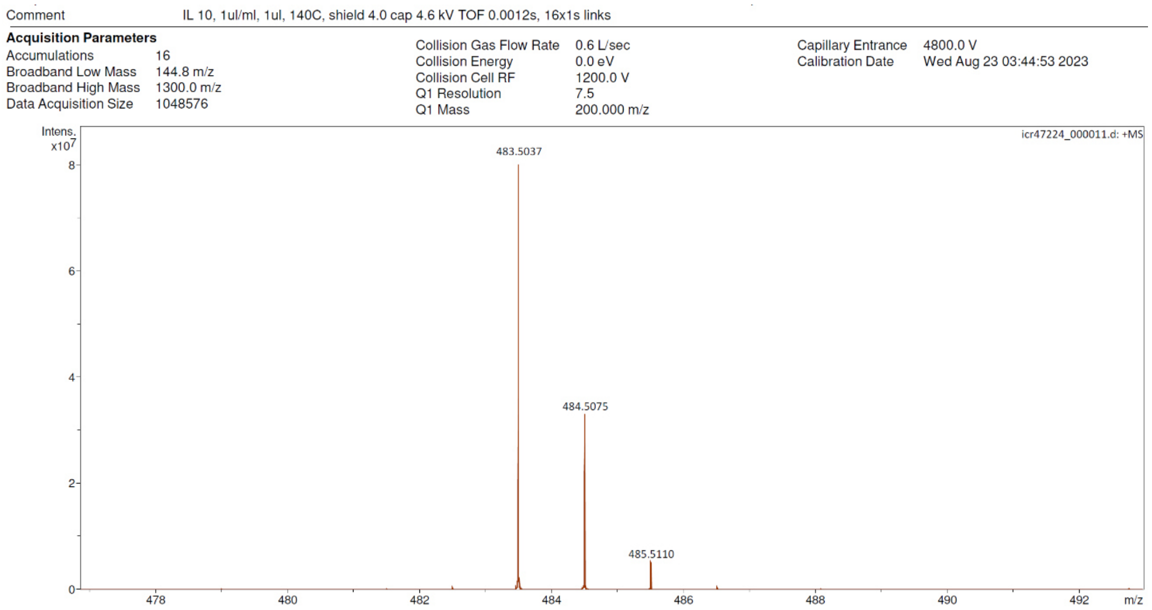

Fig. S10. Reproduction of APFD spectra of compounds already known from previous work to ensure analogous operation and at equivalent results from the new source assembly Part I: Reproduction of the APFD spectrum of Jeffamine M-2005, a basic poly(propylene glycol) being a viscous liquid at room temperature. APFD settings: shield at –3.7 kV, cap at –4.5 kV, dry gas at 1.2 l min^{-1} and 140 °C. In positive-ion APFD, Jeffamine M-2005 yielded a series of protonated molecules. The ions of the series were evenly spaced at Δ(*m/z*) = 58.0419 as expected for the C_3H_6O monomer. Ionic compositions based on accurate mass data were assigned to all peaks with a yellow mark at the top. The spectra obtained with the new setup corresponded well to earlier findings *.

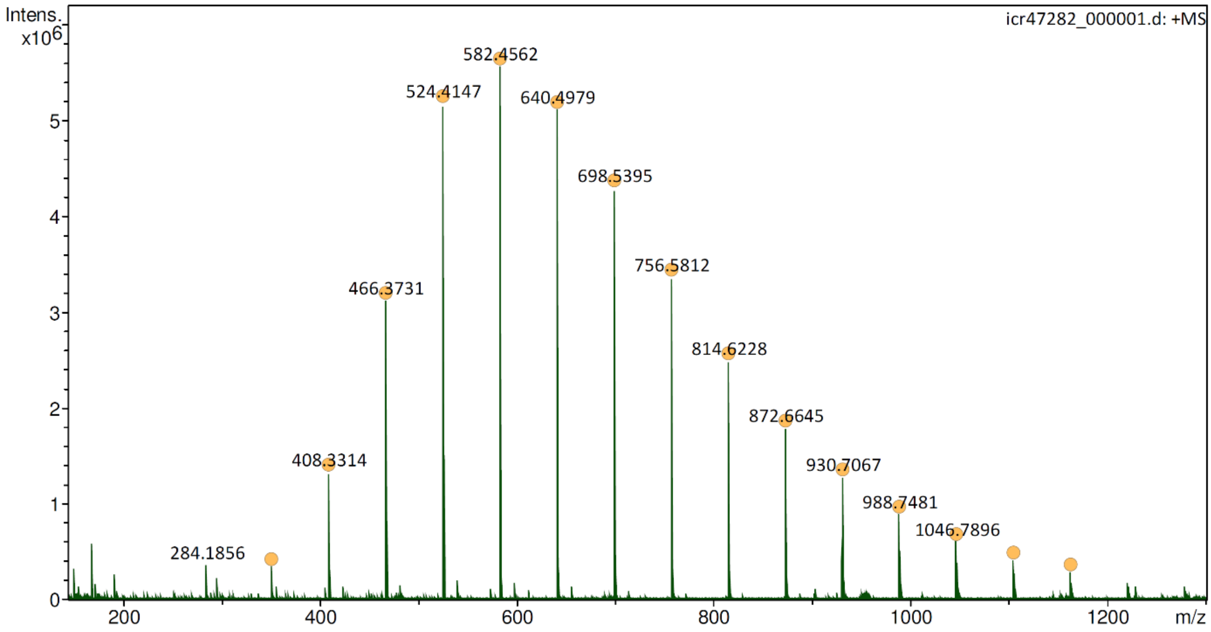

Meas. m/z	Ion Formula	m/z	err [ppm]	mSigma	rdb	e⁻ Conf
350.2898	C18H40NO5	350.2901	0.9	17.6	-0.5	even
408.3314	C21H46NO6	408.3320	1.3	6.7	-0.5	even
466.3731	C24H52NO7	466.3738	1.6	14.3	-0.5	even
524.4147	C27H58NO8	524.4157	2.0	15.9	-0.5	even
582.4562	C30H64NO9	582.4576	2.3	16.8	-0.5	even
640.4979	C33H70NO10	640.4994	2.4	14.3	-0.5	even
698.5395	C36H76NO11	698.5413	2.6	12.3	-0.5	even
756.5812	C39H82NO12	756.5832	2.6	13.7	-0.5	even
814.6228	C42H88NO13	814.6250	2.7	11.8	-0.5	even
872.6645	C45H94NO14	872.6669	2.7	11.1	-0.5	even
930.7067	C48H100NO15	930.7087	2.2	12.5	-0.5	even
988.7481	C51H106NO16	988.7506	2.6	7.1	-0.5	even
1046.7896	C54H112NO17	1046.7925	2.7	14.1	-0.5	even
1104.8321	C57H118NO18	1104.8343	2.1	33.8	-0.5	even
1162.8725	C60H124NO19	1162.8762	3.2	40.6	-0.5	even

* Gross JH. Desorption of positive and negative ions from activated field emitters at atmospheric pressure. *Eur J Mass Spectrom* 2023; 29: 21-32. DOI: 10.1177/14690667221133388.

Fig. S11. Reproduction of APFD spectra of compounds already known from previous work to ensure analogous operation and at equivalent results from the new source assembly Part II: Reproduction of the APFD spectrum of the polycyclic aromatic 1-aza-[6]helicene APFD settings: shield at –4.0 kV, cap at –4.6 kV, dry gas at 1.5 l min^{-1} and 140 °C. In positive-ion APFD, 1-aza-[6]helicene mainly yielded the $[M+H]^+$ ion at *m/z* 330.1273 due to its high proton affinity of 1000 kJ mol^{-1}. The molecular ion, $[C_{25}H_{15}N]^{+\bullet}$, *m/z* 329.1196, appeared at about 2.5 % of the $[M+H]^+$ ion intensity. Ionic compositions based on accurate mass data were assigned to all peaks with a yellow mark at the top. The spectra obtained with the new setup corresponded very well to earlier findings [§].

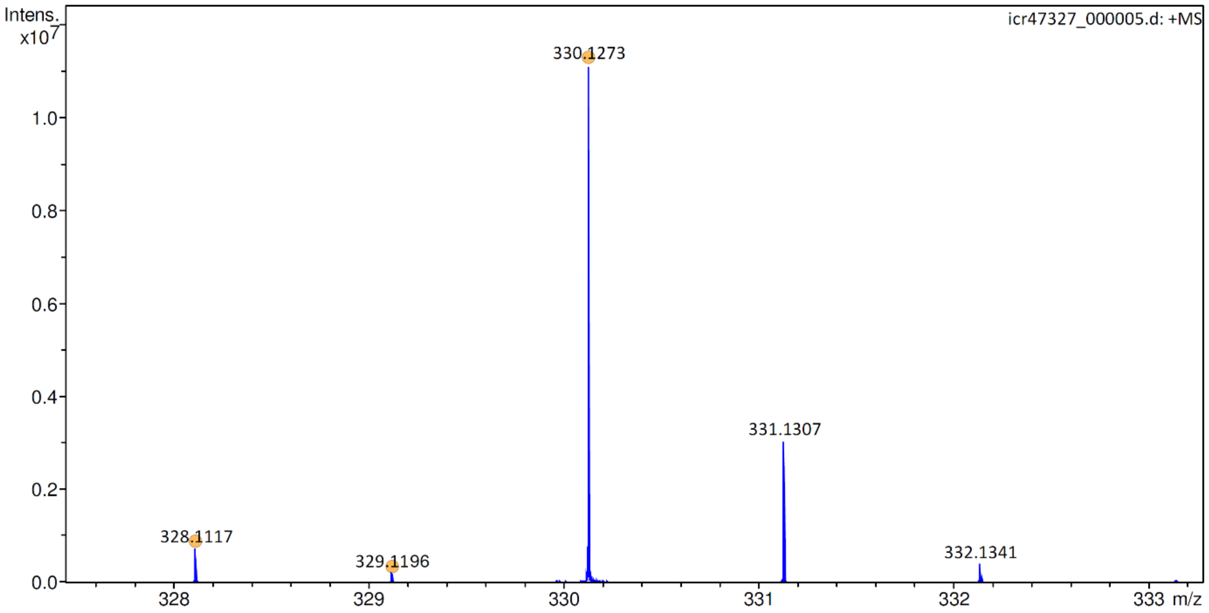

Meas. m/z	Ion Formula	m/z	err [ppm]	mSigma	rdb	e⁻ Conf	N-Rule
328.1117	C25H14N	328.1121	1.0	20.3	19.5	even	ok
329.1196	C25H15N	329.1199	1.0	720.0	19.0	odd	ok
330.1273	C25H16N	330.1277	1.2	1.8	18.5	even	ok

§ Hoyer M and Gross JH. Molecular ion formation on activated field emitters in atmospheric pressure field desorption mass spectrometry. *Anal Bioanal Chem* 2023; 415: 2307-2315. DOI: 10.1007/s00216-023-04652-4.

Fig. S12. Reproduction of APFD spectra of compounds already known from previous work to ensure analogous operation and at equivalent results from the new source assembly Part III: Reproduction of the APFD spectrum of the benzo[a]pyrene. APFD settings: shield at –4.5 kV, cap at –5.5 kV, dry gas at 1.2 l min^{-1} and 140 °C. In positive-ion APFD, benzo[a]pyrene formed the molecular ion, $[C_{20}H_{12}]^{+\bullet}$, *m/z* 252.0931. In addition, a fragment by loss of a hydrogen molecule and another signal due to some minor impurity did appear. Ionic compositions based on accurate mass data were assigned to all peaks with a yellow mark at the top. The spectra obtained with the new setup corresponded very well to earlier findings [§].

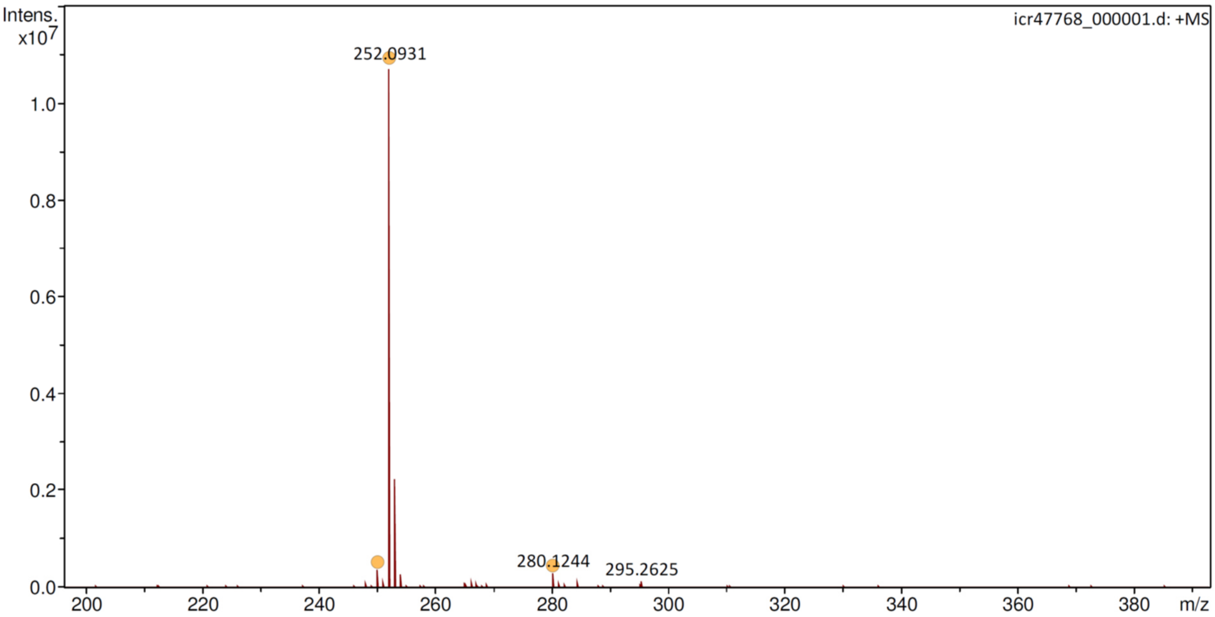

Meas. m/z	Ion Formula	m/z	err [ppm]	mSigma	rdb	e⁻ Conf	N-Rule
250.0775	C20H10	250.0777	0.9	519.6	16.0	odd	ok
252.0931	C20H12	252.0934	0.9	5.4	15.0	odd	ok
280.1244	C22H16	280.1247	0.8	15.9	15.0	odd	ok

[§] Hoyer M and Gross JH. Molecular ion formation on activated field emitters in atmospheric pressure field desorption mass spectrometry. *Anal Bioanal Chem* 2023; 415: 2307-2315. DOI: 10.1007/s00216-023-04652-4.

Fig. S13. Reproduction of APFD spectra with the new source assembly Part IV: Reproduction of the APFD spectrum of 1,1,4,4-tetraphenylbutadiene. APFD settings: shield up to –4.8 kV, cap up to –5.5 kV, dry gas at 1.2 l min^{-1} and 140 °C. In positive-ion APFD, 1,1,4,4-tetraphenylbutadiene yielded the molecular ion, $[C_{28}H_{22}]^{+\bullet}$, *m/z* 358.1709. Higher potentials improved the FI efficiency and delivered more intensive molecular ion peaks (intensities increased from 5 × 10^5 to 1.2 × 10^6 counts). The ionic composition based on accurate mass and an expanded isotopic pattern were added to the bottom spectrum. The spectra obtained with the new setup corresponded very well to earlier findings [§].

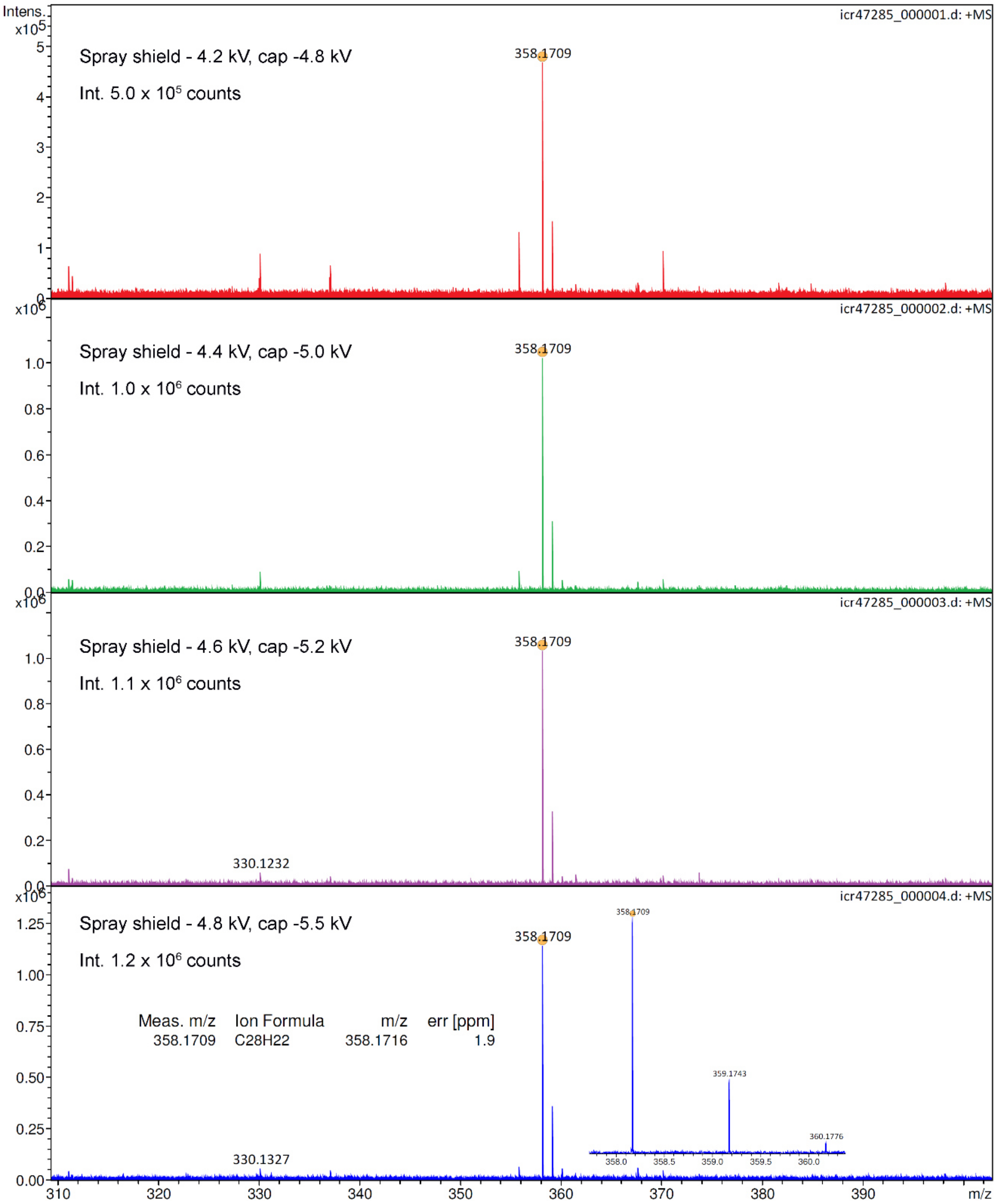

[§] Hoyer M and Gross JH. Molecular ion formation on activated field emitters in atmospheric pressure field desorption mass spectrometry. *Anal Bioanal Chem* 2023; 415: 2307-2315. DOI: 10.1007/s00216-023-04652-4.

Fig. S14. Reproduction of APFD spectra with the new source assembly Part V: Reproduction of the negative-ion APFD spectrum of Dusy Women shower gel. APFD settings: shield at +4.0 kV, cap at +5.0 kV, dry gas at 1.2 l min^{-1} and 140 °C. In this negative-ion APFD spectrum, two major series of signals corresponding to homologous ions did appear. The formulas assigned by accurate mass are supplied in the list along with the calculated *m/z* values and relative errors and corresponded to organic sulfates of increasing chain length. Yellow dots at the peak tops mark peaks with formula assignments in the list below the spectral plot. The spectrum obtained with the new setup corresponded very well to earlier findings [$]. The major ion series comprises the organic sulfates $[C_{12}H_{25}O_4S]^-$ (calc. 265.1479), $[C_{14}H_{29}O_5S]^-$ (calc. 309.1741), $[C_{16}H_{33}O_6S]^-$ (calc. 353.2003), and $[C_{18}H_{37}O_7S]^-$ (calc. 397.2265). In addition, there are sulfonates $[C_{14}H_{29}O_4S]^-$ (calc. 293.1792), $[C_{16}H_{33}O_5S]^-$ (calc. 337.2054), $[C_{18}H_{37}O_6S]^-$ (calc. 381.2316), and $[C_{20}H_{41}O_7S]^-$ (calc. 425.2578). Either series starts with a saturated aliphatic sulfate, indicating fatty acid alcohol sulfate structures. The difference to the next higher member of each ion series corresponds to C_2H_4O units, and thus, points towards a polyethyleneglycol unit being part of the structure of these ions.

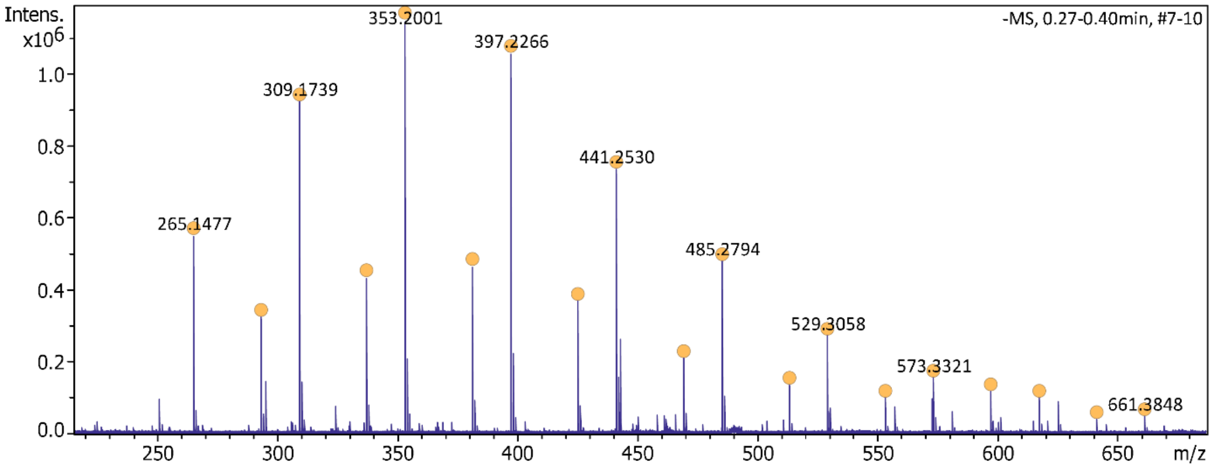

Meas. m/z	Ion Formula	m/z	err [ppm]	mSigma	rdb	e⁻ Conf	N-Rule
265.1477	C12H25O4S	265.1479	0.9	11.6	2.0	even	ok
293.1791	C14H29O4S	293.1792	0.4	13.8	2.0	even	ok
309.1739	C14H29O5S	309.1741	0.6	7.2	2.0	even	ok
337.2055	C16H33O5S	337.2054	-0.1	13.6	2.0	even	ok
353.2001	C16H33O6S	353.2003	0.5	9.0	2.0	even	ok
381.2318	C18H37O6S	381.2316	-0.5	8.2	2.0	even	ok
397.2266	C18H37O7S	397.2265	-0.2	9.6	2.0	even	ok
425.2582	C20H41O7S	425.2578	-0.7	20.3	2.0	even	ok
441.2530	C20H41O8S	441.2528	-0.6	11.9	2.0	even	ok
469.2846	C22H45O8S	469.2841	-1.1	25.1	2.0	even	ok
485.2794	C22H45O9S	485.2790	-0.9	32.7	2.0	even	ok
513.3109	C24H49O9S	513.3103	-1.1	56.4	2.0	even	ok
529.3058	C24H49O10S	529.3052	-1.1	30.0	2.0	even	ok
553.2856	C29H45O8S	553.2841	-2.9	80.3	9.0	even	ok
573.3321	C26H53O11S	573.3314	-1.1	25.3	2.0	even	ok
597.3117	C31H49O9S	597.3103	-2.3	66.0	9.0	even	ok
617.3585	C28H57O12S	617.3576	-1.4	63.3	2.0	even	ok
641.3381	C33H53O10S	641.3365	-2.5	n.a.	9.0	even	ok
661.3848	C30H61O13S	661.3838	-1.4	70.0	2.0	even	ok

[$] Gross JH. Application of atmospheric pressure field desorption for the analysis of anionic surfactants in commercial detergents. *Anal Bioanal Chem* 2023; 415: 6421-6430. DOI: 10.1007/s00216-023-04917-y.

Fig. S15. Photographs of the probe tip with the field emitter 2 mm in front of the spray shield electrode when no, moderate, and strong EHC were applied. The glow is shown in correct color and its brightness corresponds to that perceived upon visual inspection.

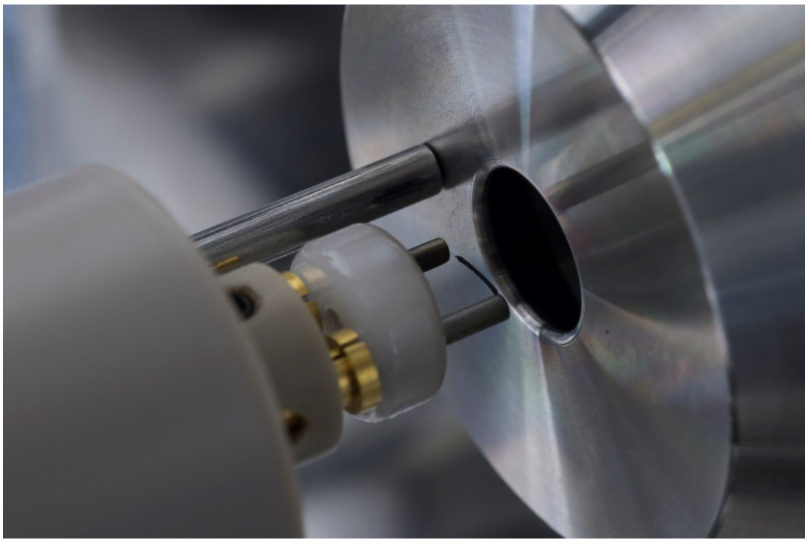

EHC off

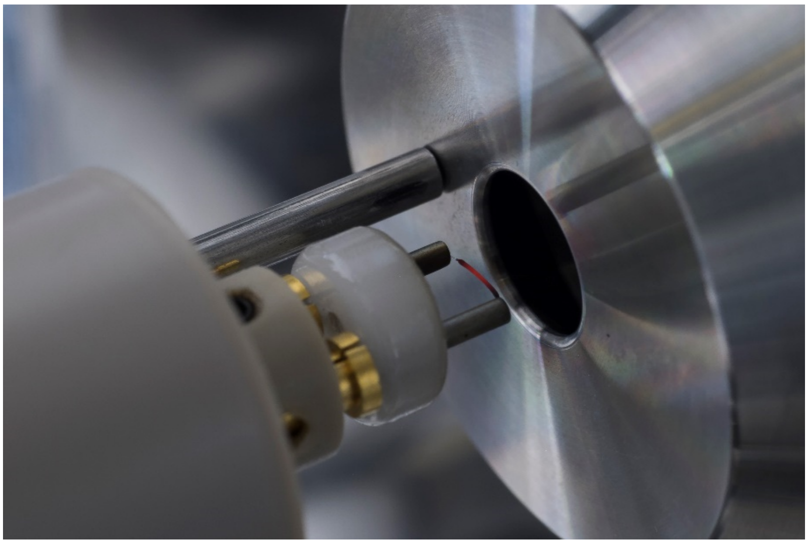

EHC 0.14 A at 1.8 V

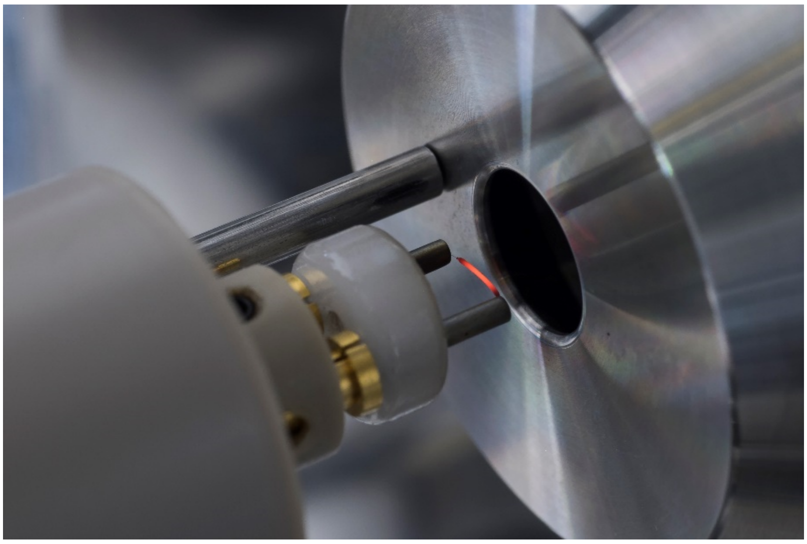

EHC 0.16 A at 2.1 V

Fig. S16. Another run of positive-ion APFD spectra of 1,1,4,4-tetraphenylbutadiene as acquired while the EHC was raised manually from 0.00 A at 0.0 V to 0.13 A at 1.5 V. The onset of desorption/ionization at 0.08 A is reflected in the base peak chromatogram (BPC). APFD settings: ion accumulation 2.0 s per transient, shield at –4.5 kV, cap at –5.5 kV, dry gas at 1.5 l min^{-1} and 140 °C. The molecular ion of 1,1,4,4-tetraphenylbutadiene, $[C_{28}H_{22}]^{+\bullet}$, *m/z* 358.1713, appeared at 0.08 A. When the EHC reached 0.10 A, an impurity, $[C_{34}H_{26}]^{+\bullet}$, *m/z* 434.2028, became more notable, thus indicating some fractionation of the sample upon heating. Yellow dots at the peak tops refer to peaks with formula assignment.

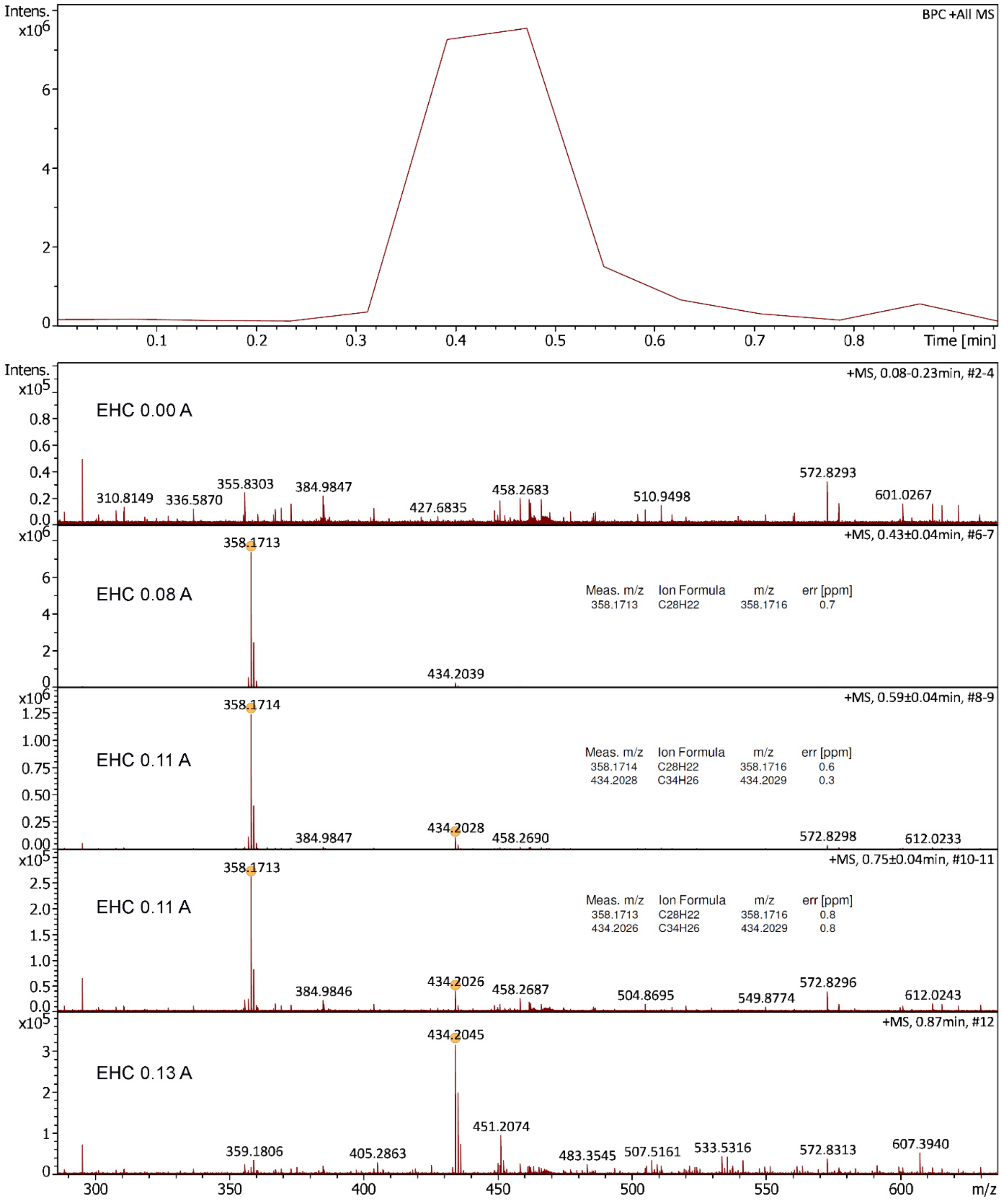

Fig. S17. Positive-ion APFD spectrum of Jeffamine M-2005 with the application of an EHC ramp up to 0.13 A. APFD settings: ion accumulation 2.0 s per transient, shield at –4.0 kV, cap at –5.0 kV, dry gas at 1.5 l min^{-1} and 140 °C. The series of protonated molecules started to appear at 0.09 A and disappeared at 0.13 A after the sample had been consumed. The effect of fractionation of the sample from low-mass to high-mass components was obvious. The spectra at the top (0.00 A) and the bottom (0.13 A) show only background noise peaks.

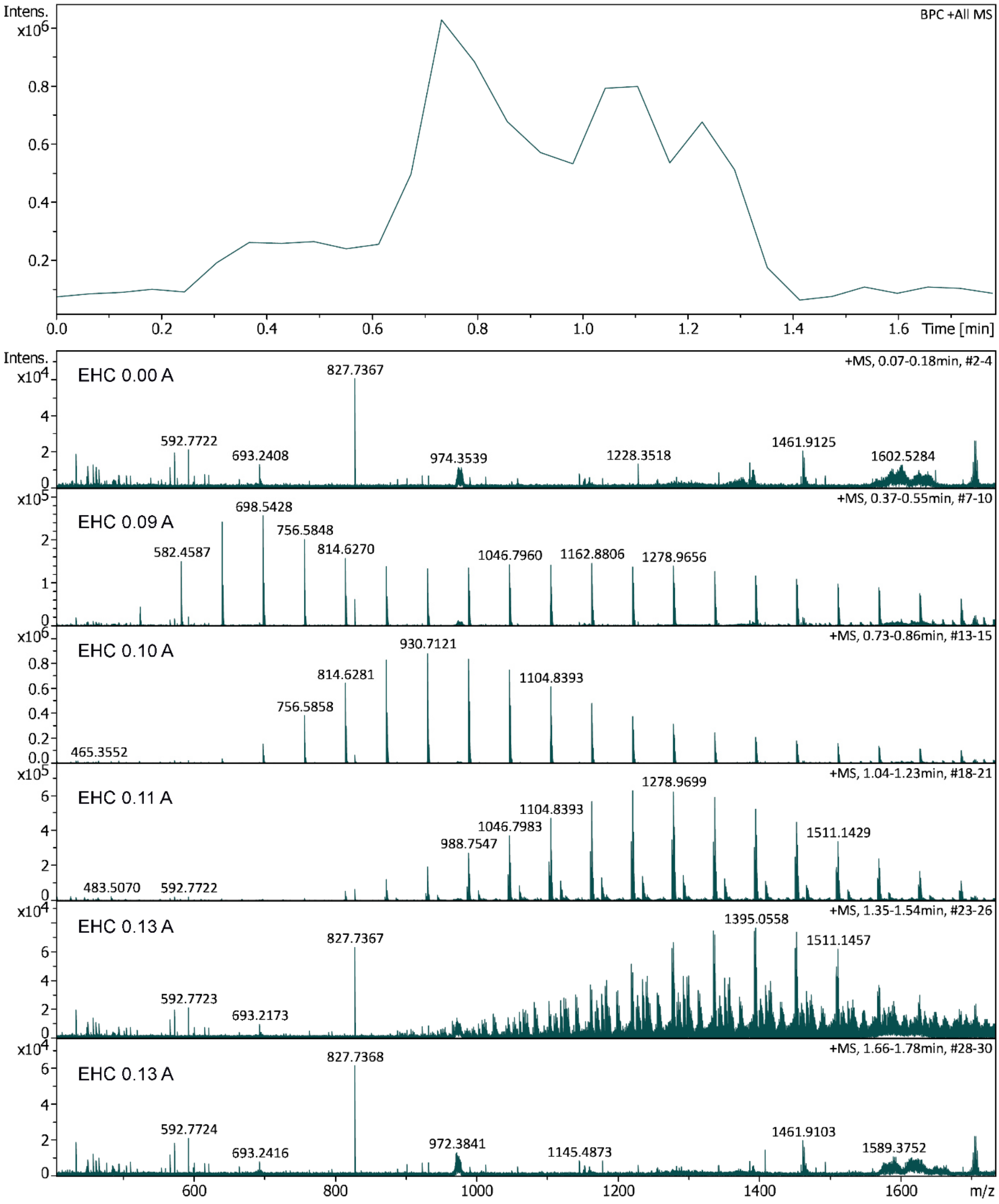

Fig. S18. Positive-ion APFD spectrum of 1 µg polystyrene of average molecular weight of 560 u obtained using an EHC ramp up to 0.13 A. APFD settings: ion accumulation 1.0 s per transient, shield at –4.8 kV, cap at –5.5 kV, dry gas at 1.5 l min^{-1} and 140 °C. The series molecular ions (even *m/z*) started to appear at 0.09 A and quickly disappeared at 0.10 A after the sample had been consumed. The series covered 6mer (*m/z* 682) to 11mer (*m/z* 1202). For more reliable formula assignment to the fragment ion peaks at odd *m/z* values, the spectrum had been internally recalibrated based on some abundant molecular ion peaks as a reference. Yellow dots at the peak tops refer to peaks with formula assignment.

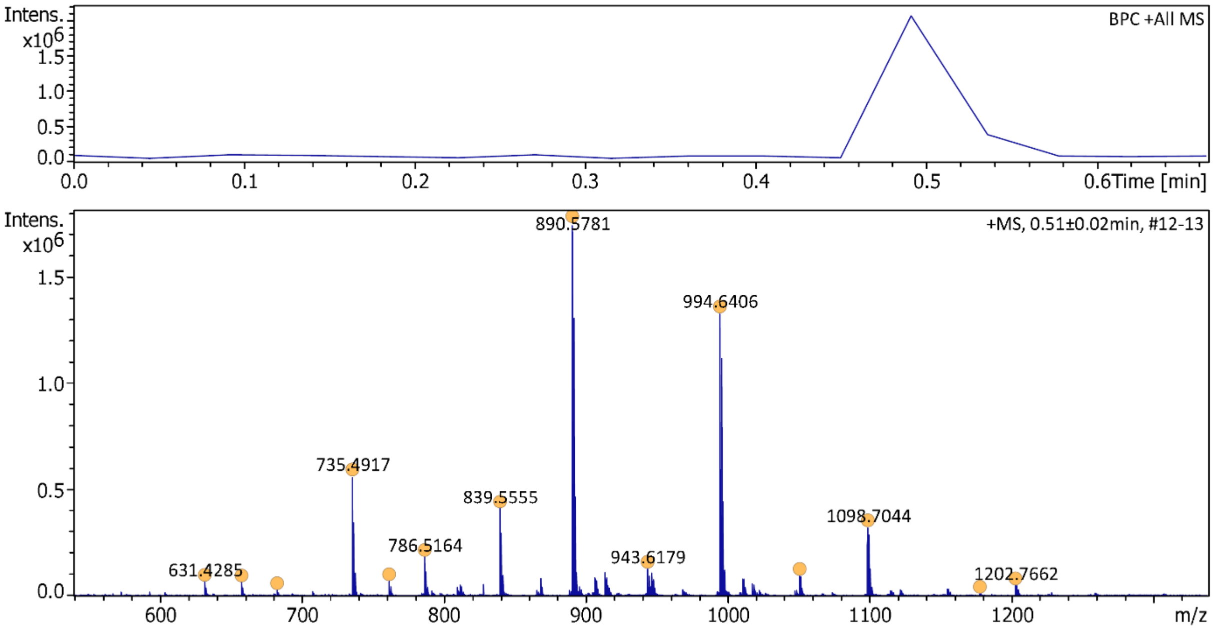

Meas. m/z	Ion Formula	m/z	err [ppm]	mSigma	rdb	e⁻ Conf	N-Rule
631.4285	C48H55	631.4298	2.0	35.3	22.0	even	ok
657.4447	C50H57	657.4455	1.1	41.4	23.0	even	ok
682.4532	C52H58	682.4533	0.2	35.3	24.5	odd	ok
735.4917	C56H63	735.4924	1.0	2.3	26.0	even	ok
761.5083	C58H65	761.5081	-0.3	10.9	27.0	even	ok
786.5164	C60H66	786.5159	-0.6	15.3	28.5	odd	ok
839.5555	C64H71	839.5550	-0.6	9.8	30.0	even	ok
890.5781	C68H74	890.5785	0.4	3.6	32.5	odd	ok
943.6179	C72H79	943.6176	-0.3	47.3	34.0	even	ok
994.6406	C76H82	994.6411	0.6	7.5	36.5	odd	ok
1050.7048	C80H90	1050.7037	-1.0	34.9	36.5	odd	ok
1098.7044	C84H90	1098.7037	-0.7	13.8	40.5	odd	ok
1177.7578	C90H97	1177.7585	0.6	71.6	43.0	even	ok
1202.7662	C92H98	1202.7663	0.1	42.7	44.5	odd	ok

Fig. S19. Positive-ion APFD spectrum of 3.5 µg polystyrene of average molecular weight of 1 ku obtained using an EHC ramp up to 0.15 A. APFD settings: ion accumulation 1.0 s per transient, shield at –4.8 kV, cap at –5.5 kV, dry gas at 1.5 l min^{-1} and 140 °C. The series molecular ions started to appear at ca. 0.09 A and disappeared at 0.13 A after the sample had been consumed. The series covered 6mer (*m/z* 682) to 16mer (*m/z* 1724). For more reliable formula assignment to the fragment ion peaks, the spectrum had been internally recalibrated based on several abundant PS molecular ion peaks as a reference. Yellow dots at the peak tops refer to peaks with formula assignment.

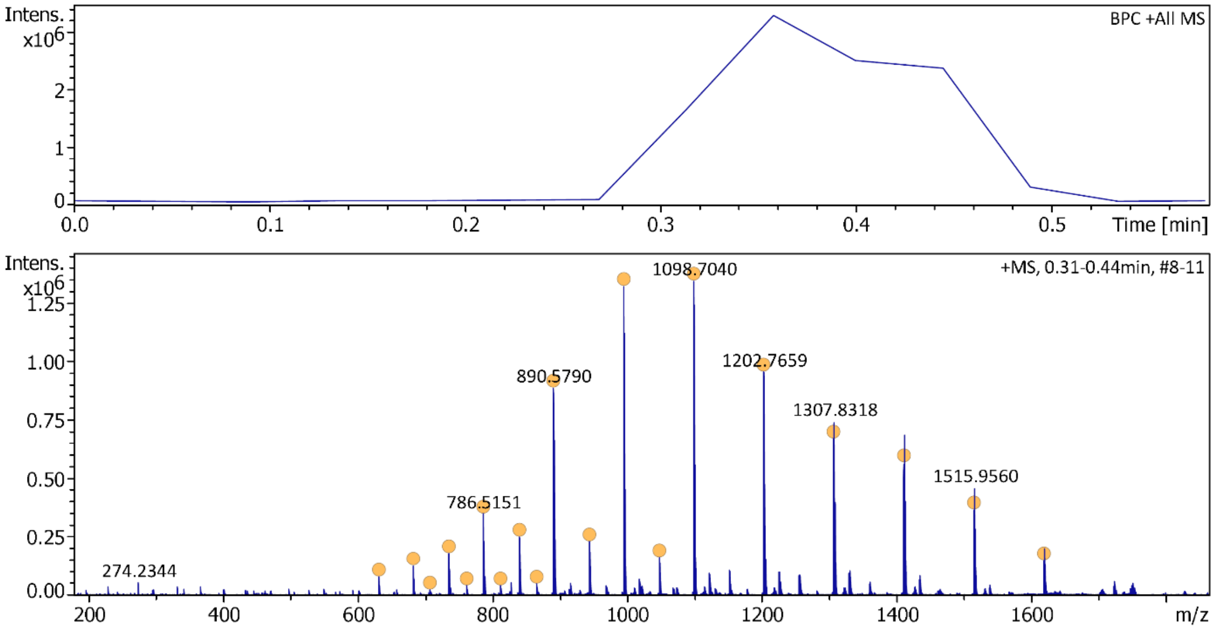

Meas. m/z	Ion Formula	m/z	err [ppm]	mSigma	rdb	e⁻ Conf	N-Rule
631.4306	C48H55	631.4298	-1.2	10.9	22.0	even	ok
682.4535	C52H58	682.4533	-0.3	3.6	24.5	odd	ok
707.4622	C54H59	707.4611	-1.5	39.4	26.0	even	ok
735.4943	C56H63	735.4924	-2.6	9.2	26.0	even	ok
761.5108	C58H65	761.5081	-3.6	24.0	27.0	even	ok
786.5151	C60H66	786.5159	1.0	10.6	28.5	odd	ok
811.5257	C62H67	811.5237	-2.4	9.5	30.0	even	ok
839.5566	C64H71	839.5550	-1.9	10.2	30.0	even	ok
865.5737	C66H73	865.5707	-3.4	11.5	31.0	even	ok
890.5790	C68H74	890.5785	-0.5	5.2	32.5	odd	ok
943.6190	C72H79	943.6176	-1.5	12.6	34.0	even	ok
994.6414	C76H82	994.6411	-0.3	7.2	36.5	odd	ok
1047.6838	C80H87	1047.6802	-3.4	16.2	38.0	even	ok
1098.7040	C84H90	1098.7037	-0.3	3.6	40.5	odd	ok
1202.7659	C92H98	1202.7663	0.3	3.0	44.5	odd	ok
1306.8286	C100H106	1306.8289	0.2	6.6	48.5	odd	ok
1410.8925	C108H114	1410.8915	-0.7	7.9	52.5	odd	ok
1514.9521	C116H122	1514.9541	1.3	5.7	56.5	odd	ok
1619.0221	C124H130	1619.0167	-3.3	12.6	60.5	odd	ok

Fig. S20. Positive-ion APFD spectrum of 1,1,4,4-tetraphenylbutadiene obtained using the timsTOFflex instrument as depicted in Figs. S5–S8. Compare to Fig. S16 for the corresponding FT-ICR spectrum. APFD settings: ion accumulation 1.0 s per spectrum, shield at –5.0 kV, dry gas at 4.0 l min^{-1} and 150 °C, and EHC ramp up to 0.10 A.

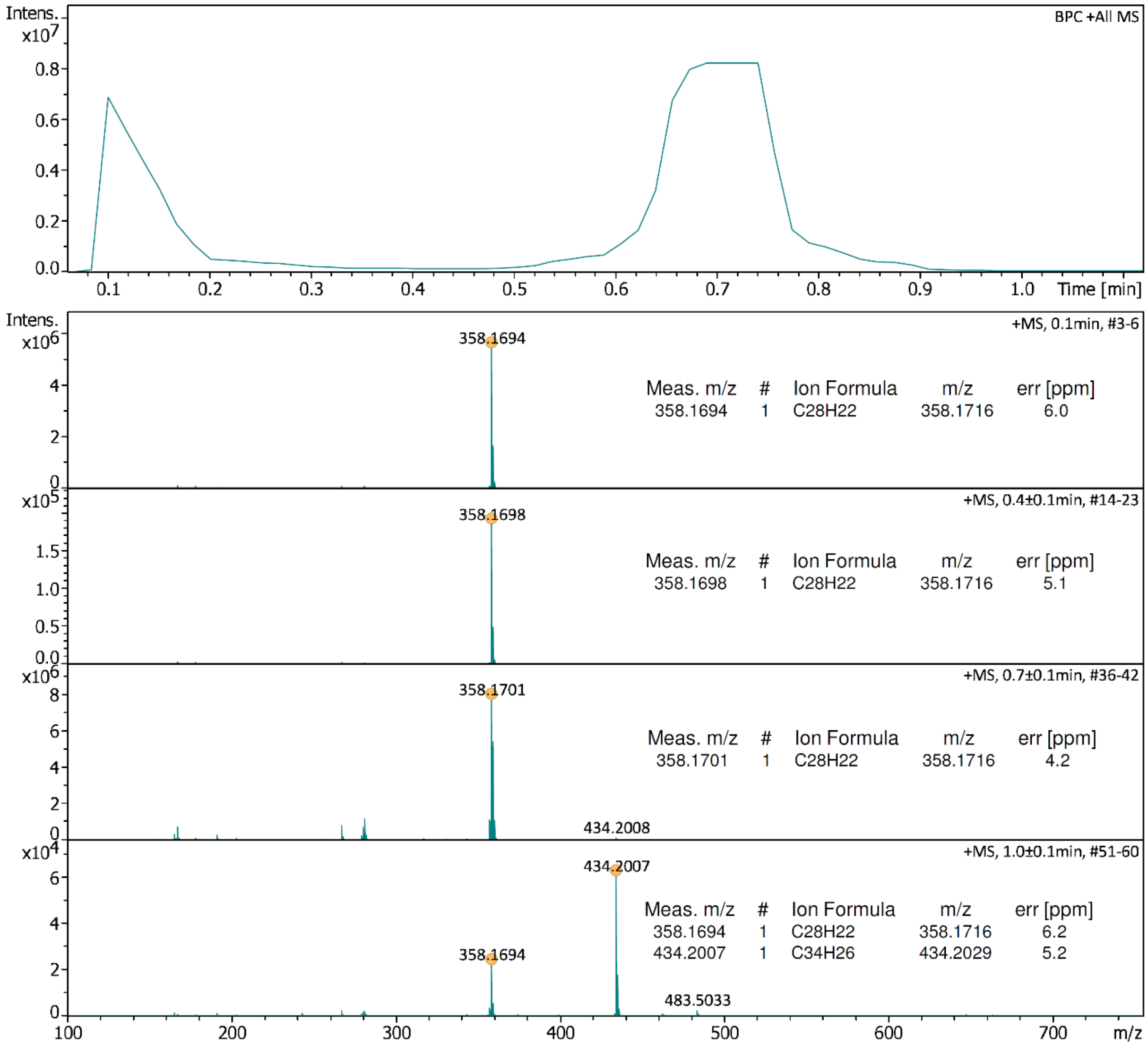

Fig. S21. Positive-ion APFD spectrum of fluoranthene obtained using the timsTOFflex instrument as depicted in Figs. S5–S8. Compare to Fig. 7 of the article for the corresponding FT-ICR spectrum. APFD settings: ion accumulation 1.0 s per spectrum, shield at –5.0 kV, dry gas at 2.0 l min^{-1} and 150 °C, and EHC ramp up to 0.10 A. It is noteworthy that the timsTOFflex instrument already detected the molecular ion from the beginning (#3–24) at an intensity of about 3×10^4 counts while the signal reached an almost two orders of magnitude higher level of 4×10^6 counts during the most active desorption period (#32–33). In the last spectrum (#37–44), a residual amount of 1,1,4,4-tetraphenylbutadiene appeared in addition (Fig. S20).

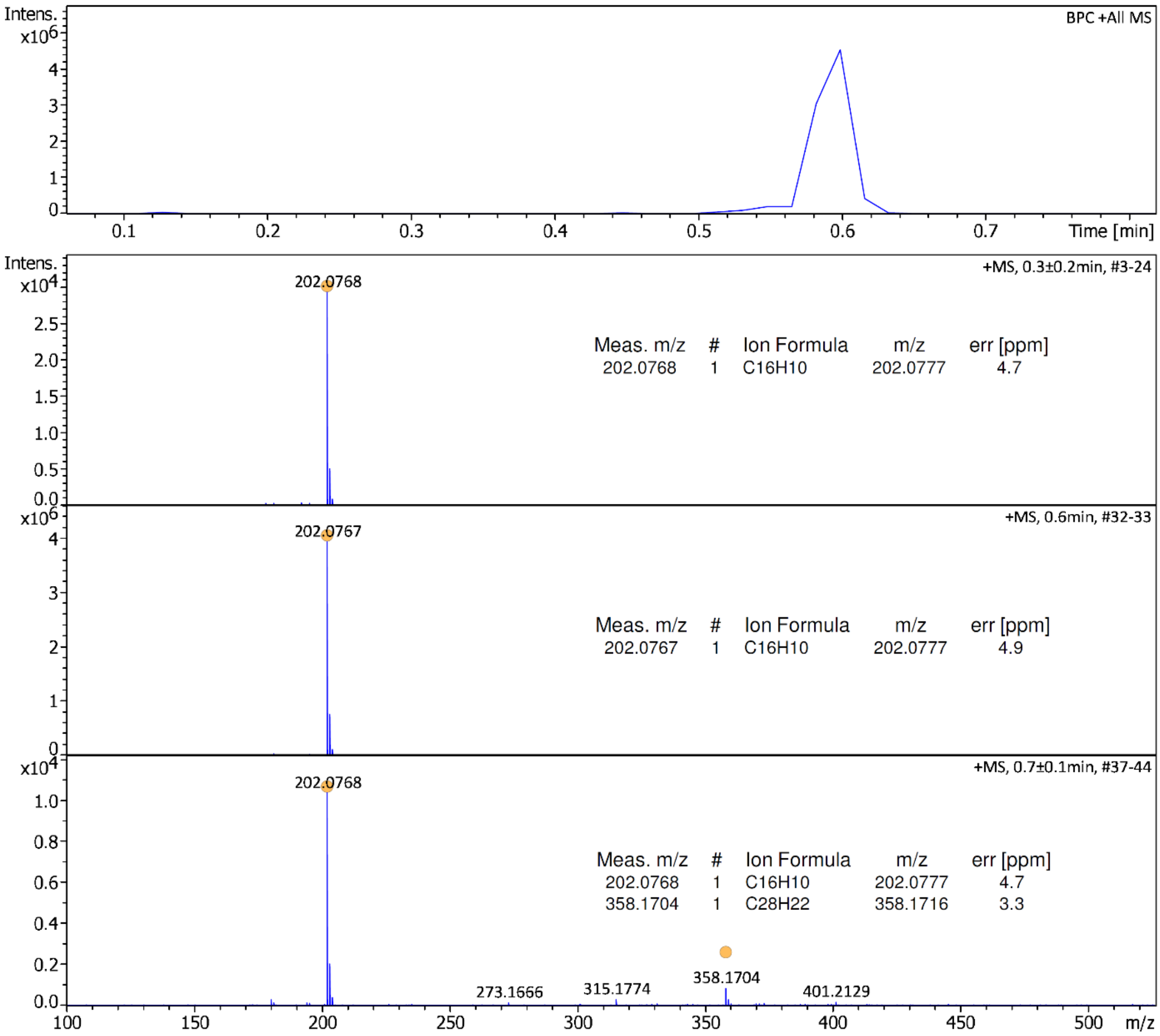

Fig. S22. Positive-ion APFD spectrum of 3.5 µg polystyrene of average molecular weight of 1 ku obtained using the timsTOFflex instrument as depicted in Figs. S5–S8.
APFD settings: ion accumulation 1.0 s per spectrum, shield at –4.8 kV, dry gas at 4.0 l min^{-1} and 150 °C, and EHC ramp up to 0.16 A. The series molecular ions also started to appear at ca. 0.09 A and disappeared at 0.14 A after the sample had been consumed. The series covered 6mer (*m/z* 682) to 17mer (*m/z* 1724). In contrast to the FT-ICR spectrum (Fig. S19), there is a slightly lower level of fragmentation but also a shift to a somewhat lower average molecular weight, which must not necessarily reflect the APFD process but could also be attributed to differences in ion transfer parameters between the two instruments.

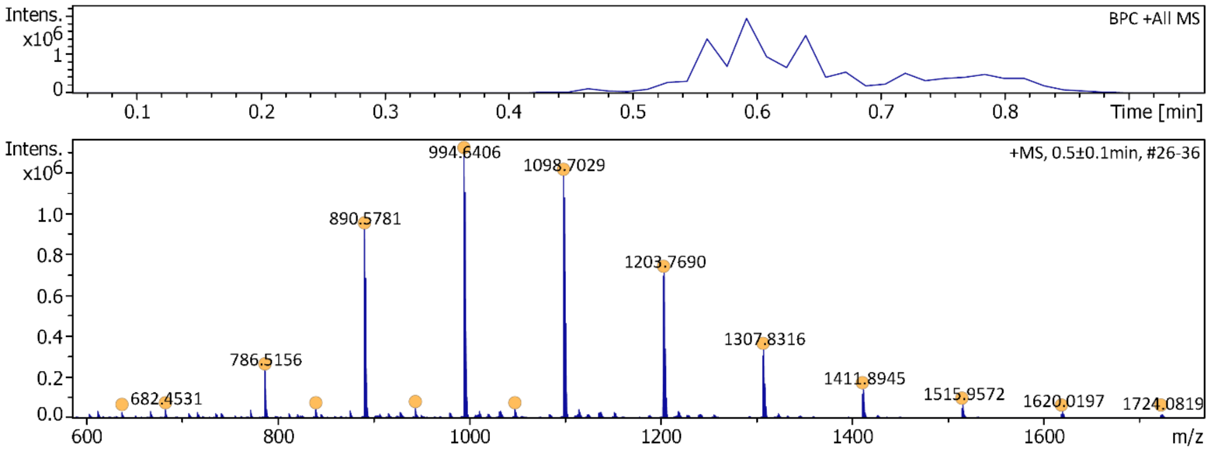

Meas. m/z	#	Ion Formula	m/z	err [ppm]	mSigma	#mSigma	Score	rdb	e⁻ Conf	N-Rule
637.3828	1	C49H49	637.3829	0.1	39.7	1	100.00	26.0	even	ok
682.4531	1	C52H58	682.4533	0.4	39.0	1	100.00	24.5	odd	ok
786.5156	1	C60H66	786.5159	0.3	22.3	1	100.00	28.5	odd	ok
839.5549	1	C64H71	839.5550	0.2	30.5	1	100.00	30.0	even	ok
890.5781	1	C68H74	890.5785	0.5	6.1	1	100.00	32.5	odd	ok
943.6169	1	C72H79	943.6176	0.8	50.6	1	100.00	34.0	even	ok
994.6406	1	C76H82	994.6411	0.5	13.2	1	100.00	36.5	odd	ok
1047.6793	1	C80H87	1047.6802	0.9	87.9	1	100.00	38.0	even	ok
1098.7029	1	C84H90	1098.7037	0.7	14.1	1	100.00	40.5	odd	ok
1202.7655	1	C92H98	1202.7663	0.7	16.6	1	100.00	44.5	odd	ok
1306.8280	1	C100H106	1306.8289	0.7	20.7	1	100.00	48.5	odd	ok
1410.8910	1	C108H114	1410.8915	0.3	21.8	1	100.00	52.5	odd	ok
1514.9535	1	C116H122	1514.9541	0.4	32.2	1	100.00	56.5	odd	ok
1619.0160	1	C124H130	1619.0167	0.4	46.8	1	100.00	60.5	odd	ok
1723.0786	1	C132H138	1723.0793	0.4	45.6	1	100.00	64.5	odd	ok

Fig. S23. Another run of the negative-ion APFD spectrum of Dusy Women shower gel acquired using the timsTOFflex instrument (cf. Fig. S14 for the spectrum using the ApexQe). APFD settings: ion accumulation 1.0 Hz, shield at 4.3 kV, dry gas at 4.0 l min^{-1} and 150 °C, and EHC ramp up to 0.14 A. Signals of low intensity were detected right from the beginning while the major desorption/ionization occurred upon gentle heating. The bottom spectrum shows the sum of the acquisition along with a formula list.

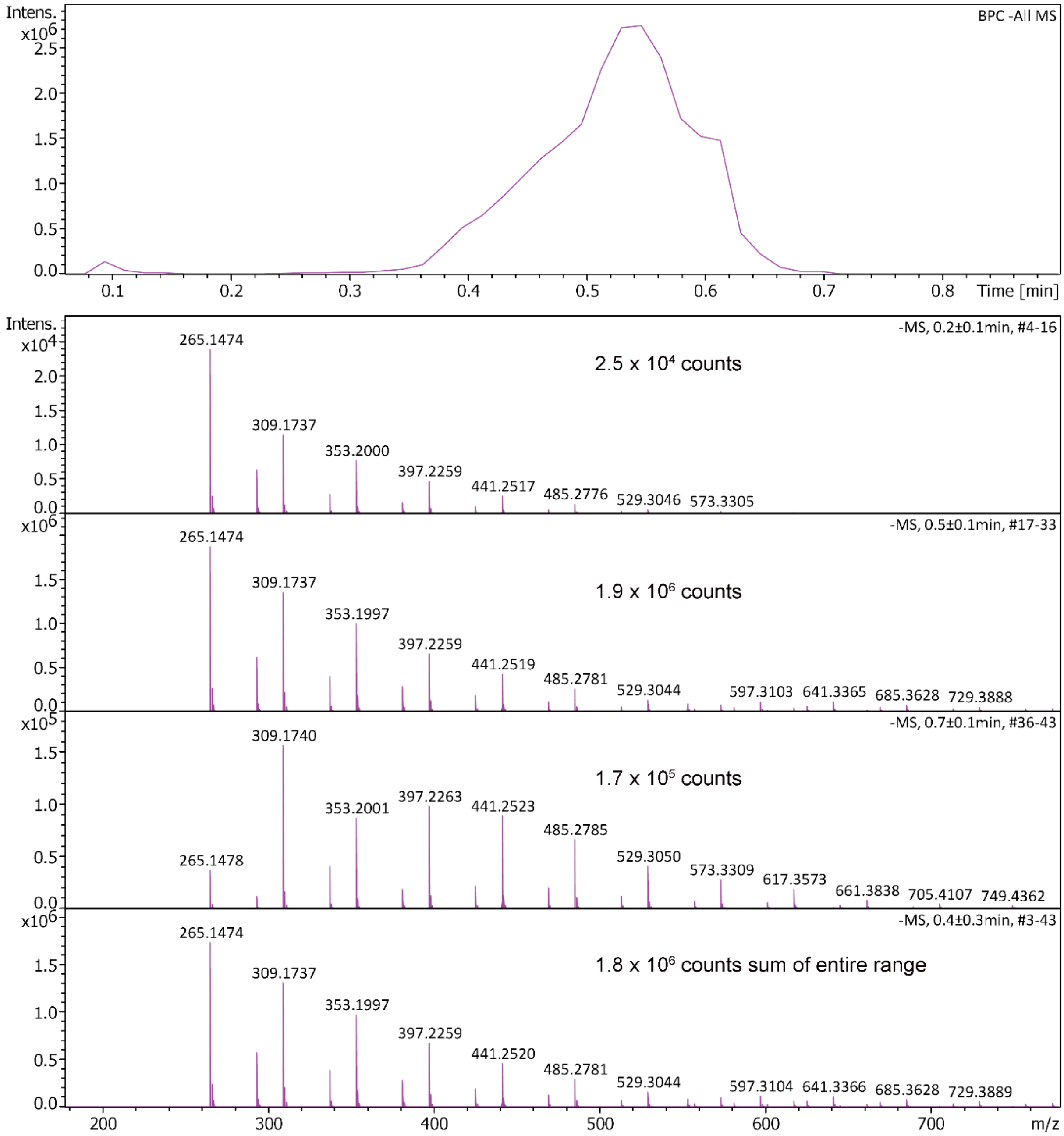

Meas. m/z	#	Ion Formula	m/z	err [ppm]
265.1474	1	C12H25O4S	265.1479	1.8
279.1642	1	C13H27O4S	279.1636	-2.3
293.1788	1	C14H29O4S	293.1792	1.5
309.1737	1	C14H29O5S	309.1741	1.4
337.2049	1	C16H33O5S	337.2054	1.5
353.1997	1	C16H33O6S	353.2003	1.7
381.2310	1	C18H37O6S	381.2316	1.6
397.2259	1	C18H37O7S	397.2265	1.6
425.2571	1	C20H41O7S	425.2578	1.8
441.2520	1	C20H41O8S	441.2528	1.8
469.2832	1	C22H45O8S	469.2841	1.8
485.2781	1	C22H45O9S	485.2790	1.7
513.3096	1	C24H49O9S	513.3103	1.4
529.3044	1	C24H49O10S	529.3052	1.4
553.2842	1	C29H45O8S	553.2841	-0.2
573.3306	1	C26H53O11S	573.3314	1.4
597.3104	1	C31H49O9S	597.3103	-0.1

Fig. S24. Negative-ion APFD spectrum of 0.2 µg of perfluorononanoic acid (PFNA) acquired using the timsTOFflex instrument. APFD settings: ion accumulation 1.0 Hz, shield at 4.3 kV, dry gas at 4.0 l min^{-1} and 150 °C, and EHC ramp up to 0.14 A. The spectrum shows the [M–H]$^{-}$ ion, $[C_9F_{17}O_2]^{-}$, *m/z* 462.9627, a fragment by loss of CO_2, $[C_8F_{17}]^{-}$, *m/z* 418.9728, and the [2M–H]$^{-}$ cluster ion, $[C_{18}HF_{34}O_4]^{-}$, *m/z* 926.9324.

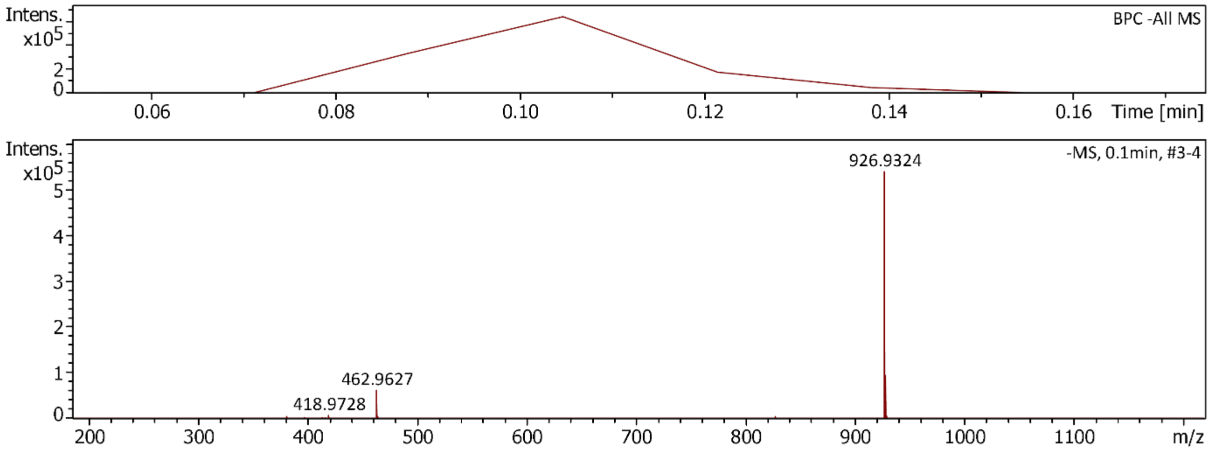

Meas. m/z	#	Ion Formula	m/z	err [ppm]	mSigma	#mSigma	Score	rdb	e⁻ Conf	N-Rule
418.9728	1	C8F17	418.9734	1.4	n.a.	1	100.00	0.0	even	ok
462.9627	1	C9F17O2	462.9632	1.2	22.2	1	100.00	1.0	even	ok
926.9324	1	C18HF34O4	926.9337	1.5	15.7	1	80.89	1.0	even	ok
